# Research on contextual sentiment recognition based on neural encoding and decoding and knowledge guidance

**DOI:** 10.1038/s41598-026-52490-y

**Published:** 2026-05-14

**Authors:** Xiangyu Cheng

**Affiliations:** Department of Industrial Engineering and Operations Research, University of California, Berkeley, CA 94720 USA

**Keywords:** Information systems and information technology, Mathematics and computing

## Abstract

Contextual sentiment recognition is critical for applications such as intelligent customer service and mental health monitoring. However, existing models struggle with multimodal heterogeneity, knowledge scarcity, and inadequate capture of dynamic emotional transitions. To address these challenges, we propose a dual-branch neural encoding–decoding architecture integrated with dynamic knowledge guidance. The model processes multimodal features (text, speech, video) and contextual dependencies through separate branches, incorporating both explicit knowledge (personality traits, domain rules) and implicit knowledge distilled from large language models. A dynamic context window adapts based on emotional shifts to enhance real-time perception. Experiments on IEMOCAP, MELD, and DailyDialog datasets demonstrate that our full model achieves accuracies of 82.1%, 78.3%, and 76.2%, respectively, surpassing state-of-the-art benchmarks including fine-tuned GPT-4. The lightweight version (18.2 M parameters) maintains high inference speed (950 samples/sec) while reducing deployment costs. Furthermore, the model exhibits strong cross-dataset generalization and practical utility. This work provides an efficient framework that effectively addresses core challenges in contextual sentiment recognition, balancing performance with practicality for real-world deployment.

## Introduction

In recent years, contextual sentiment recognition technology has become a core supporting capability in the fields of intelligent interaction and mental health. The global market for intelligent customer service has expanded significantly, with a growing expectation from users for systems to perceive emotional changes in conversations^1^. Meanwhile, the digital transformation in the mental health sector is accelerating, as many online psychological counseling platforms are beginning to require real-time analysis of users’ emotional states to adjust intervention strategies^2,3^. The volume of multimodal dialogue data, including text, speech, and video, has seen substantial year-on-year growth, while the accuracy of traditional unimodal sentiment recognition remains limited, making it difficult to meet the needs of complex scenarios^4^. The core of contextual sentiment recognition lies in capturing the dynamic evolution of emotions from continuous conversations, rather than analyzing the sentiment polarity of individual sentences in isolation—for example, a user’s dissatisfaction in a customer service conversation may stem from unresolved issues in the previous round, and a user’s silence in a mental health conversation may imply anxiety. However, existing technologies still have deficiencies in handling multimodal heterogeneous data, integrating domain knowledge, and capturing long-distance contextual dependencies, which limit the generalization ability of models in real-world scenarios.

Intelligent customer service is a typical application scenario of contextual sentiment recognition. For example, when a user repeatedly asks the same question, if the system responds based solely on the neutral sentiment of the current sentence, it may overlook the user’s underlying irritability^5,6^; whereas a contextual sentiment recognition model can capture the user’s emotional changes and proactively adjust response strategies, such as transferring to human customer service or providing a more detailed solution. Mental health monitoring is another important application area. Patients’ emotional expressions in online conversations are often subtle—for example, I haven’t been sleeping well recently may imply a tendency towards depression, which requires comprehensive judgment based on information such as decreased interest and decreased energy from previous conversations^7^. Contextual sentiment recognition models can analyze the sentiment trends in conversations in real time, such as continuous negative sentiment growth over multiple rounds, and promptly trigger an early warning mechanism to help counselors adjust intervention plans and provide more personalized care for patients.

Despite significant progress in recent years, existing approaches to contextual sentiment recognition face three interrelated technical challenges that fundamentally limit their performance and generalization capability. First, multimodal heterogeneous fusion remains a challenge. Real-world conversations involve text (semantic content), speech (prosody), and video (facial expressions and gestures), each residing in fundamentally different feature spaces. Simple concatenation or weighted fusion often leads to information conflict or redundancy, failing to capture complementary signals. Second, knowledge scarcity limits generalization. Current models are trained on narrow datasets and lack access to: (1) explicit domain knowledge (e.g., speaker traits, domain-specific sentiment rules); and (2) commonsense knowledge about emotional concepts. Third, models inadequately capture dynamic emotional transitions. Emotions evolve and shift across dialogue turns, requiring both long-range context and fine-grained local sensitivity. RNNs suffer from vanishing gradients beyond 10 turns, while Transformers face high computational cost (threefold increase beyond 20 turns) and rigid context windows unable to adapt to shifting emotional dynamics.

These three challenges—multimodal heterogeneity, knowledge scarcity, and inadequate dynamic context capture—are not independent. Their interactions compound the difficulty of contextual sentiment recognition. For example, multimodal fusion becomes more challenging when knowledge about speaker-specific expression patterns is unavailable, and dynamic context modeling requires both well-aligned multimodal features and knowledge-guided attention to accurately track emotional trajectories.

The primary objective of this study is to develop a dual-branch neural encoding and decoding architecture to address the core challenges of heterogeneous modal fusion, knowledge scarcity, and inadequate dynamic context capture. To provide a clear framework for evaluating our proposed innovations, we formulate the following three Research Questions (RQs), each corresponding to a key contribution of this work:

RQ1 (dual-branch architecture): does the proposed dual-branch neural encoding–decoding architecture, which processes multimodal features and contextual dependencies in parallel, effectively address the challenge of heterogeneous modal fusion? Specifically, does it outperform single-modality baselines and standard multimodal fusion approaches?

RQ2 (knowledge guidance mechanism): does the dynamic knowledge guidance mechanism, integrating both explicit knowledge (personality traits, domain rules) and implicit knowledge (distilled from large language models), compensate for knowledge scarcity and improve model generalization to unfamiliar scenarios?

RQ3 (adaptive context window): does the adaptive context window, which dynamically adjusts its size based on emotional shift intensity, successfully balance long-range dependency capture with fine-grained emotional transition modeling, thereby improving recognition accuracy in multi-turn conversations?

**Fig. 1:**
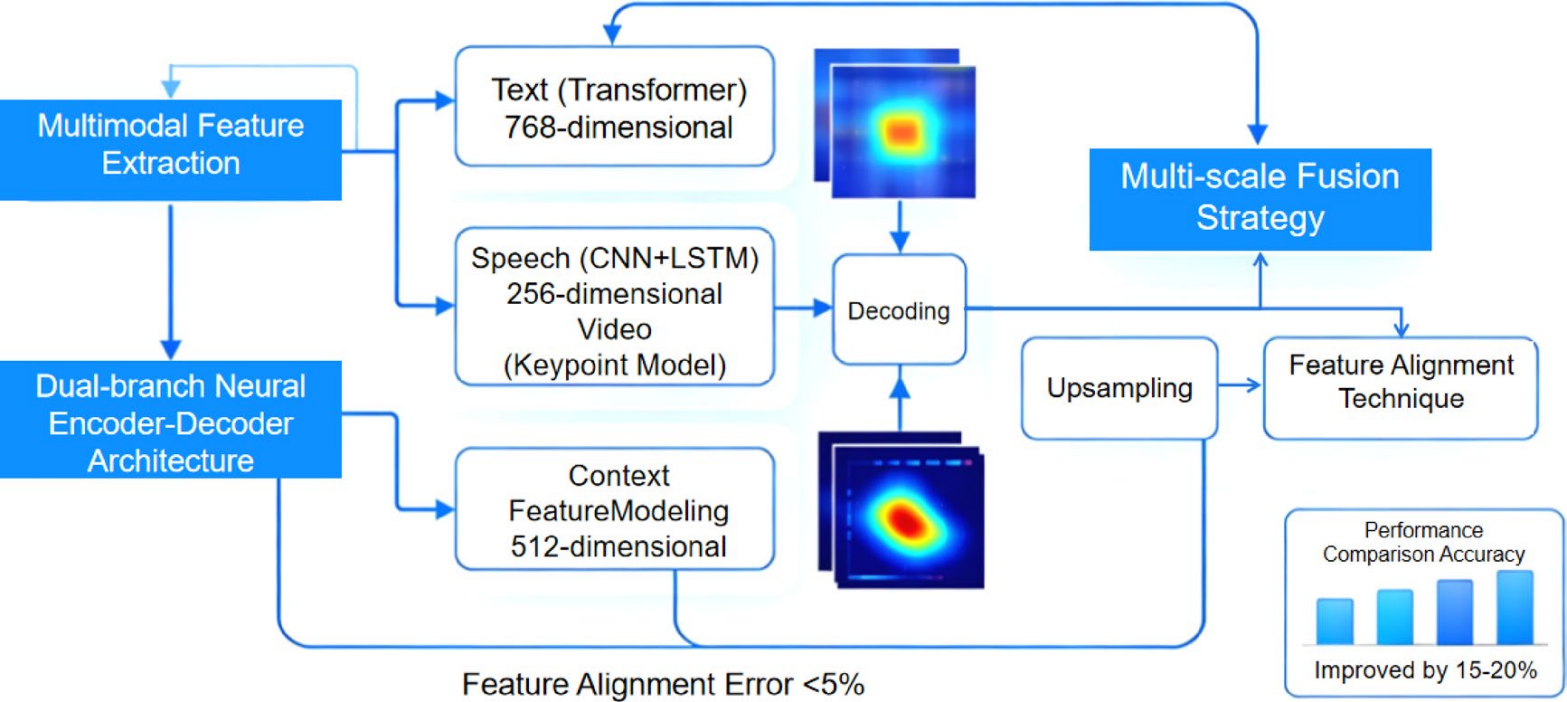
Schematic diagram of research objectives.

A schematic diagram illustrating the research objectives is shown in Fig. 1.

To address these interconnected challenges, we propose a dual-branch neural encoding–decoding architecture integrated with dynamic knowledge guidance. Our approach makes the following key contributions:

1. *Dual-branch neural encoding–decoding architecture* we propose a novel architecture that separately processes multimodal features (text, speech, video) and contextual dependencies through parallel branches, effectively addressing the challenge of heterogeneous modal fusion. The multimodal branch employs atrous spatial pyramid pooling (ASPP) with multiple dilation rates to capture features at different granularities, while the contextual branch uses Transformer encoders to model cross-turn emotional dependencies.
2. *Dynamic knowledge guidance mechanism* we introduce a knowledge integration framework that combines both explicit knowledge (speaker personality traits encoded via MLP, domain-specific sentiment rules via embedding) and implicit knowledge distilled from large language models (via KL divergence loss). An attention mechanism dynamically adjusts knowledge influence weights based on conversational context, compensating for knowledge scarcity and improving generalization to unfamiliar scenarios.
3. * Adaptive context window* we design a dynamic context window that adjusts its size based on the intensity of emotional shifts—expanding to capture long-range dependencies during stable emotional periods (W = 10) and contracting to focus on local transitions during rapid emotional changes (W = 3). This mechanism balances the need for both long-term context and fine-grained emotional dynamics.
4. *Comprehensive evaluation and practical validation* extensive experiments on three benchmark datasets (IEMOCAP, MELD, DailyDialog) demonstrate that our full model achieves state-of-the-art performance (82.1%, 78.3%, and 76.2% accuracy, respectively), outperforming strong baselines including fine-tuned GPT-4. Ablation studies validate the contribution of each core module. Furthermore, we provide a lightweight version (18.2 M parameters) with high inference speed (950 samples/sec) and validate the model’s practical utility through cross-dataset generalization tests and an e-commerce customer service deployment case study.

The detailed technical implementation of these innovations, including specific network architectures, attention mechanisms, and loss functions, is presented in Section "Contextual sentiment recognition model integrating neural encoding and decoding with knowledge guidance". Their effectiveness is empirically validated through comprehensive experiments in Section "Experimental verification and analysis".

The structure of this article is arranged as follows: Section "Related work" reviews the current research progress and core challenges in contextual sentiment recognition, focusing on neural encoding–decoding, knowledge guidance, and dynamic context modeling. Section "Theoretical Foundations" introduces the relevant theoretical foundations, covering neural encoding–decoding techniques, knowledge guidance methods, and contextual sentiment modeling. Section "Contextual sentiment recognition model integrating neural encoding and decoding with knowledge guidance" proposes a contextual sentiment recognition model that integrates neural encoding–decoding with knowledge guidance, detailing its overall architecture, encoder-decoder modules, knowledge guidance mechanisms, and loss function design. Section "Experimental verification and analysis" presents experimental verification and analysis, including experimental setup, result analysis, module effectiveness, generalization verification, and engineering application value. Section "Discussion" discusses the model’s contributions, limitations, and future research directions. Finally, the conclusions summarize the main findings and contributions of this study.

## Related work

### Current research progress

Existing research focuses on three main areas: neural encoding and decoding, knowledge guidance, and contextual sentiment recognition. These areas form a multidimensional technical system. Neural encoding and decoding architectures are widely used in sentiment recognition. The encoder extracts multimodal features, such as text, speech, and video, using Transformer or convolutional neural networks, while the decoder adopts a multi-scale fusion strategy to integrate feature information from different levels. For instance, based on the U-Net structural design, high-level semantic features output by the encoder are aligned and fused with low-level detailed features to enhance the expressive power of sentiment features^8–10^. In terms of knowledge guidance, both explicit knowledge injection and implicit knowledge distillation are commonly adopted. Explicit knowledge includes speaker personality traits and sentiment-triggering rules within the domain, while implicit knowledge is obtained by transferring the sentiment-understanding ability of large language models. Regarding contextual sentiment recognition, research focuses on capturing temporal context using recurrent neural networks or Transformer architectures to model sentiment dependencies across time, while combining multimodal context fusion methods to perceive dynamic changes in sentiment^11–13^.

Research into neural encoding and decoding has further refined the design of multi-scale and hierarchical structures. The encoder extracts features of different scales via modules such as dilated spatial pyramid pooling, and the decoder restores emotional detail information via upsampling and feature concatenation^14^. Some models also introduce a dual-branch encoder structure that processes modal and contextual features separately, enhancing the specificity of feature extraction. In terms of knowledge guidance, explicit knowledge injection methods offer greater flexibility^15^. For instance, after encoding personality traits as vectors, the weights of these traits in the decoder can be dynamically adjusted via an attention mechanism^16^. This enables the model to adapt its emotional judgement logic according to different speakers^17,18^. Implicit knowledge distillation involves designing a specialised loss function that enables small models to learn the emotional probability distribution of larger models, thereby improving the model’s ability to generalise. Regarding contextual sentiment recognition, the cross-modal context modelling method has been optimised. A cross-attention mechanism captures associations between text, speech, and video, while the Transformer’s self-attention mechanism addresses long-distance contextual dependencies. Some models also introduce dynamic context windows that adjust the scope of the context according to the dialogue’s length and emotional changes, thereby improving sentiment recognition accuracy further^19,20^. While these advancements have provided a solid technical foundation for contextual sentiment recognition, there are still limitations when dealing with complex scenarios^21,22^.

Beyond general contextual sentiment recognition, a more granular challenge lies in handling ‘multi-aspect’ scenarios where different entities within the same utterance may exhibit contradictory sentiment polarities. This is particularly prevalent in domains such as product reviews and customer service dialogues. Wang et al. proposed C3DA, a contrastive cross-channel data augmentation framework that generates diverse training samples by swapping aspect-related sentiment expressions across channels, thereby improving model robustness against fine-grained sentiment conflicts. Such contrastive augmentation strategies are complementary to our dynamic context modeling and knowledge guidance mechanisms, offering potential pathways for further enhancing fine-grained sentiment perception.

In comparison, the research directions outlined above each have their own complementary strengths and limitations^23–25^. Although neural encoder-decoder architectures (such as those inspired by U-Net) perform exceptionally well in multi-scale feature extraction, their focus on single-modal processing pipelines often makes it difficult to integrate heterogeneous modal features. Knowledge-guided methods, while capable of effectively injecting domain expertise through explicit rules or implicit knowledge distillation, typically lack the ability to dynamically adapt to conversational context, treating knowledge as static input rather than context-modulated guidance. Context-based sentiment recognition models, though capable of capturing temporal dependencies across dialogue turns, face challenges in balancing long-range dependencies with fine-grained sentiment transitions, particularly when using fixed context windows^26,27^. These complementary limitations—insufficient heterogeneous fusion, static knowledge integration, and rigid context modeling—prompted us to propose a dual-branch architecture with dynamic knowledge guidance. This architecture simultaneously addresses these shortcomings through parallel multimodal-context processing and adaptive knowledge weight allocation.

### Core pain points

One of the core challenges in contextual sentiment recognition is the difficulty of multimodal heterogeneous fusion. There are fundamental differences in the feature spaces of different modalities—text features capture discrete semantic information, speech features encode continuous prosodic dynamics, and video features represent spatiotemporal facial expressions and gestures. Models that employ simple feature concatenation or weighted fusion strategies often struggle to leverage the complementary advantages of multimodality, as information conflict and redundancy can degrade representation quality^28–31^. This challenge is well-documented in the multimodal sentiment analysis literature, where achieving effective cross-modal alignment remains an open research problem^32–34^.

Another key challenge is the limited ability to capture contextual dependencies. Emotional expressions in conversations exhibit strong temporal dependencies^35,36^. When traditional recurrent neural networks are used to process conversations exceeding 10 rounds, their accuracy in capturing long-range emotional dependencies declines, and they may even fail to effectively capture cross-round emotional associations^37,38^. Although Transformer models can address the long-range issue, their computation time increases by more than threefold when conversations exceed 20 rounds, making it difficult to meet the requirements of real-time interaction^39,40^. Furthermore, dynamic changes in context also increase the difficulty of recognition. Because these models cannot flexibly adjust their focus, this leads to biases in emotion recognition^41–43^. These limitations directly impact the effectiveness of contextual emotion recognition models in real-world scenarios^44^.

The three core issues identified in the present study—modal heterogeneous fusion difficulty, knowledge scarcity, and weak contextual dependency capture—directly correspond to the experimental validations presented in Section "Experimental setup". The multimodal fusion challenge is specifically evaluated through comparative experiments on the IEMOCAP dataset, where the dual-branch architecture of the present study demonstrates significant improvements over single-modality baselines^45,46^. The issue of knowledge scarcity is systematically addressed in the context of ablation studies, with the objective of quantifying the contribution of explicit and implicit knowledge integration^47,48^. The validity of the contextual dependency problem is confirmed through cross-dataset generalization experiments, in which dynamic context windows demonstrate particular effectiveness in long conversation scenarios. The alignment between the challenges identified and empirical validation ensures that the experimental design directly targets the fundamental limitations in existing research^49,50^.

To provide a clear picture of the current research landscape and to identify the specific gaps that motivate our work, Table 1 summarizes representative methods for contextual sentiment recognition across three benchmark datasets. The comparison is organized by method type, ranging from recurrent and attention-based models to large language model fine-tuning and knowledge-augmented approaches.

**Table 1:** Comparison of representative methods for contextual sentiment recognition.

Method	Key approach	Datasets used	Reported accuracy (%)	Key limitations/gaps
DialogRNN (2019)	RNN with GRU, speaker-state tracking	IEMOCAP, MELD	68.2/65.1/63.5	Vanishing gradients beyond 10 turns; no explicit multimodal fusion; static context window
MGAT (2021)	Multimodal graph attention, cross-modal edges	IEMOCAP, MELD	72.3/66.4/64.8	Graph construction heuristic; limited long-range dependency; no knowledge integration
CAT (2021)	Co-attention for text-speech fusion	IEMOCAP, MELD	73.5/67.2/65.7	Only two modalities; fixed co-attention layers; no personality/domain knowledge
EMO-BART (2022)	BART fine-tuned with emotion prompts	IEMOCAP, MELD, DailyDialog	76.9/72.3/70.5	Single-modality (text) only; no temporal context modeling beyond BART’s max length
MultiEMO (2023)	Multi-task learning (emotion + sentiment)	IEMOCAP, MELD, DailyDialog	80.5/76.1/74.2	Task interference; no explicit knowledge guidance; fixed context
KGAN (2023)	Knowledge graph augmentation + hierarchical fusion	IEMOCAP, MELD, DailyDialog	79.3/75.6/73.4	Static knowledge injection; no dynamic context window; heavy graph computation
LLaMA-7B-finetuned (2023)	Fine-tuned large language model (7B)	IEMOCAP, MELD, DailyDialog	75.8/70.5/68.9	High resource cost; no multimodal input (text-only); overfitting on small datasets
GPT-4-finetuned (2023)	Fine-tuned GPT-4 via API	IEMOCAP, MELD, DailyDialog	79.2/75.1/73.0	Proprietary; no video/speech input; high latency; expensive
ISNet (2024)	Individual speaker normalization + speech-only	IEMOCAP	79.2	Unimodal (speech only); no context modeling across turns
Our Model (Full)	Dual-branch neural encoding–decoding + dynamic knowledge guidance + adaptive context window	IEMOCAP, MELD, DailyDialog	82.1/78.3/76.2	–

As shown in Table 1, existing methods exhibit three common limitations that persist across different architectural families. First, multimodal heterogeneity is poorly addressed: most models (DialogRNN, EMO-BART, LLaMA, GPT-4) rely on single-modality text or speech, while those that attempt multimodal fusion (MGAT, CAT) use simple concatenation or co-attention without explicit alignment mechanisms for heterogeneous feature spaces. Second, knowledge integration remains static: KGAN injects knowledge graphs but lacks dynamic, context-aware modulation of knowledge influence; other methods ignore external knowledge altogether, leading to poor generalization on unseen domains. Third, context windows are rigid: almost all models use fixed-length context (e.g., 5, 10, or 20 turns), failing to adapt to the variable pace of emotional shifts—long windows introduce noise during rapid transitions, while short windows miss long-range dependencies. These gaps directly motivate our dual-branch architecture (separate processing of multimodal features and temporal context), dynamic knowledge guidance (attention-modulated knowledge weighting), and adaptive context window (size adjusted by emotional shift intensity).

3. **Theoretical foundations**

This section provides the essential theoretical background that underpins our proposed model. Rather than offering exhaustive derivations, we focus on the core principles—self-attention mechanisms, knowledge distillation, and temporal context modeling—that directly inform the architectural decisions presented in Section "Contextual sentiment recognition model integrating neural encoding and decoding with knowledge guidance".

### Self-attention and transformer architecture

The self-attention mechanism forms the foundation of modern sequence modeling approaches. It enables models to capture dependencies between different positions within a sequence by computing attention weights. The core computation is expressed as.

1 $$Attention\left( {Q,K,V} \right) = soft\max \left( {\frac{{QK^{T} }}{{\sqrt {d_{k} } }}} \right)V$$

where *Q, K,* and *V* represent query, key, and value matrices obtained through linear transformations of input features, $QK^{T}$ computes pairwise similarity scores between all queries and keys and *d*_*k*_ is the dimension of keys that scales the dot product to prevent excessively large values. This equation computes the scaled dot-product attention used in our knowledge-guided fusion and Transformer encoders. It is executed once per attention layer per forward pass. *Q, K,* and *V* are linear projections of input features; the softmax produces attention weights that sum to 1. The scaling factor $\sqrt {d_{k} }$ uses $d_{k}$=512 (hidden dimension of the contextual encoder). This value is standard in Transformer implementations and was not tuned. The importance of attention is validated by removing the knowledge-guided attention module, which reduces IEMOCAP accuracy by 3.6%. The multi-head variant (not separately formulated) uses 8 heads as specified in Eq. (10).

In the context of sentiment recognition, this mechanism allows the model to weigh the importance of different modalities and temporal positions when constructing emotional representations. For instance, when processing a conversation, self-attention can assign higher weights to utterances that carry strong emotional cues while discounting neutral statements, effectively capturing cross-turn emotional dependencies.

Multi-head attention extends this capability by performing multiple attention operations in parallel:

2 $${\mathrm{MultiHead}}\left( {{\mathrm{Q}},K,V} \right){\text{ = Concat}}\left( {{\mathrm{head}}_{1} , \ldots ,{\mathrm{head}}_{{\mathrm{h}}} } \right){\mathrm{W}}^{{\mathrm{O}}}$$

where each ${\mathrm{head}}_{{\mathrm{i}}} = {\mathrm{Attention}}\left( {{\mathrm{QW}}_{{\mathrm{i}}}^{{\mathrm{Q}}} , K{\mathrm{W}}_{{\mathrm{i}}}^{{\mathrm{K}}} , V{\mathrm{W}}_{{\mathrm{i}}}^{{\mathrm{V}}} } \right)$ learns different aspects of feature interactions, ${\mathrm{W}}^{{\mathrm{O}}}$ is the output projection matrix that combines concatenated head outputs This is particularly valuable for multimodal sentiment analysis, as different heads can specialize in cross-modal relationships (e.g., aligning facial expressions with speech prosody) or temporal dynamics (e.g., tracking emotional escalation across turns).

The Transformer encoder stacks multiple self-attention layers with feed-forward networks and layer normalization, enabling the extraction of hierarchical representations. The positional encoding mechanism injects sequence order information, which is crucial for understanding the temporal flow of conversations.

### Knowledge distillation principles

Knowledge distillation provides a framework for transferring the capabilities of large, complex models (teachers) to smaller, more efficient models (students). The fundamental principle involves training the student to mimic the teacher’s output distribution rather than solely optimizing on ground-truth labels.

The distillation loss is typically formulated using Kullback–Leibler (KL) divergence:

3 $$L_{kd} { = }\mathop \sum \limits_{{\mathrm{i}}} {\mathrm{KL}}\left( {{\mathrm{softmax}}\left( {\frac{{{\mathrm{z}}_{{\mathrm{t}}} }}{{\uptau }}} \right){\text{; softmax}}\left( {\frac{{{\mathrm{z}}_{{\mathrm{s}}} }}{{\uptau }}} \right)} \right)$$

where, $L_{kd}$ is the knowledge distillation loss that measures how well the student model mimics the teacher’s output distribution, ${\mathrm{z}}_{{\mathrm{t}}}$ and ${\mathrm{z}}_{{\mathrm{s}}}$ are the logits of teacher and student models, and ${\uptau }$ is a temperature parameter that controls the smoothness of the probability distribution. Higher temperatures produce softer distributions, revealing inter-class relationships that provide richer training signals than hard labels. This loss is computed during training to transfer sentiment understanding from GPT-4 (teacher) to our model (student). It is added to the total loss with weight λ_1_ = 0.5. The temperature τ = 2 was selected via grid search, where τ = 2 outperformed τ = 1 and τ = 3 by 1.8% and 2.6%, respectively. Ablation without distillation loss reduces average accuracy by 2.6%, confirming its contribution.

The temperature parameter plays a critical role in distillation effectiveness. When ${\uptau }$ = 1, the distribution reflects the original class probabilities. As ${\uptau }$ increases, the distribution becomes more uniform, exposing the relative similarities between classes. For sentiment recognition, this means the student can learn not just that an utterance is “angry,” but also that anger is more similar to frustration than to happiness—knowledge that is valuable for nuanced emotion understanding.

Beyond output distribution matching, feature-based distillation transfers intermediate representations from teacher to student, aligning their hidden states at selected layers. This is particularly relevant for multimodal sentiment analysis, where different layers may capture modality-specific or fused emotional features.

### Temporal context modeling in conversations

Conversational sentiment understanding requires modeling dependencies across multiple utterances. Unlike isolated sentence analysis, contextual sentiment recognition must capture how emotions evolve, persist, or shift throughout a dialogue.

Recurrent neural networks (RNNs) and their variants (LSTM, GRU) provide a foundational approach to sequence modeling by maintaining hidden states that propagate information across time steps:

4 $$h_{t} = \phi \left( {{\mathrm{x}}_{{\mathrm{t}}} {\mathrm{,h}}_{{\text{t - 1}}} } \right)$$

where, $\phi \left( \cdot \right)$ is an activation function (e.g., tanh or ReLU), ${\mathrm{x}}_{{\mathrm{t}}}$ represents the features of utterance ${\mathrm{t}}$, and ${\mathrm{h}}_{{\mathrm{t}}}$ encodes the conversational context up to that point. However, RNNs struggle with long-range dependencies due to vanishing gradients, limiting their effectiveness in multi-turn conversations.

Transformer-based architectures address this limitation through self-attention, which provides direct connections between any two positions in the sequence. The attention mechanism computes context-aware representations as:

5 $$h_{t} = \mathop \sum \limits_{j = 1}^{T} \alpha_{tj} x_{j}$$

where $\alpha_{tj}$ are attention weights that determine how much utterance $t$ should be paid to utterance are,$x_{j}$ is the feature representation of utterance $j$. This enables the model to capture both local emotional transitions (e.g., immediate reactions) and long-range dependencies (e.g., recurring emotional themes across a conversation).

Dynamic context modeling extends this concept by recognizing that optimal context length may vary with conversational dynamics. During periods of emotional stability, longer context helps establish baseline emotional states and track gradual trends. During rapid emotional shifts, focusing on recent utterances becomes more important for capturing fine-grained transitions. This principle motivates adaptive context mechanisms that adjust their temporal scope based on the intensity of emotional changes.

The emotional intensity between consecutive utterances can be quantified as:

6 $$\Delta e_{t} = \left| {e_{t} - e_{t - 1} } \right|$$

where $e_{t}$ represents the emotional state at turn $t$. Larger values of $\Delta e_{t}$ indicate emotional shifts, suggesting the need for more localized attention, while smaller values indicate stability, where broader context may be beneficial. This equation computes the emotional shift intensity that drives the adaptive context window. It is evaluated at each turn $t$ during both training and inference. $e_{t}$ is the predicted emotional state (the softmax probability of the predicted class before thresholding). The necessity of dynamic window adjustment is validated by the “No dynamic window” ablation, where removing the adaptive mechanism reduces IEMOCAP accuracy by 4.8%.

### Problem formulation

Task definition. We formulate contextual emotion recognition as a supervised sequence classification problem. Let a conversation consist of T utterances U = [u_1, u2_,…, uT], where each utterance *u*_*t*_ is associated with three modality inputs: text ${\mathrm{x}}_{{\mathrm{t}}}^{{{\mathrm{text}}}}$, speech (acoustic) ${\mathrm{x}}_{{\mathrm{t}}}^{{{\mathrm{speech}}}}$, and video ${\mathrm{x}}_{{\mathrm{t}}}^{{{\mathrm{video}}}}$. For each utterance *u*_*t*_, the model must predict a single emotion label y_t_ ∈ Y, where Y is a discrete set of emotion categories.

Input–output mapping. The model defines a function f:(x,${\mathrm{x}}_{{\mathrm{t}}}^{{{\mathrm{speech}}}}$,${\mathrm{x}}_{{\mathrm{t}}}^{{{\mathrm{video}}}}$, H_<t_) → ${\hat{\mathrm{y}}}_{{\mathrm{t}}}$, where:

X_text_ = [${\mathrm{x}}_{1}^{{{\mathrm{text}}}}$ t,…,${\mathrm{x}}_{T}^{{{\mathrm{text}}}}$], similarly for speech and video;

H_<t_ = {y_1_,…, yt_−1_} denotes the previous ground-truth emotion labels (available during training) or previously predicted labels (during inference), representing the conversational context.

Objective function. The model is trained to minimize a composite loss over the training set D:$\mathop {\min }\limits_{\theta } \frac{1}{\left| D \right|}\mathop \sum \limits_{ \in D} \mathop \sum \limits_{t = 1}^{T} \left( {L_{cls} \left( {y_{t} ,\hat{y}_{t} } \right) + \lambda_{1} L_{KD} \left( {\hat{y}_{t}^{student} ,\hat{y}_{t}^{teacher} } \right) + \lambda_{2} L_{align} \left( {x_{t}^{text} ,x_{t}^{speech} ,x_{t}^{video} } \right)} \right)$, where $\theta$ denotes all trainable parameters, ${\mathrm{L}}_{{{\mathrm{cls}}}}$ is the class-weighted cross-entropy loss, ${\mathrm{L}}_{{{\mathrm{KD}}}}$ is the knowledge distillation loss (from GPT-4 teacher), and ${\mathrm{L}}_{{{\mathrm{align}}}}$ is the modality alignment loss. The hyperparameters ${\uplambda}_{{1}}$ and ${\uplambda}_{{2}}$ control the contribution of each auxiliary loss.

Constraints. The model operates under the following constraints:

1. Context window constraint: The effective context length W is dynamic but bounded: 3 ≤ W ≤ 10, adjusted based on emotional shift intensity Δe_t_.
2. Modality availability constraint: During training, we simulate missing modalities with probability pdrop = 0.2 per modality; during inference, any modality may be missing, and the model must produce a prediction using available modalities only.
3. Causality constraint: At inference time, the model cannot access future utterances; predictions for u_t_ depend only on u_1_,…, ut and their corresponding modalities.

Terminology clarification: Sentiment vs. emotion. In the literature, “sentiment recognition” often refers to polarity classification (positive/negative/neutral), while “emotion recognition” refers to discrete categorical states (e.g., anger, joy, sadness). The datasets used in this work (IEMOCAP, MELD, DailyDialog) provide discrete emotion labels, making this an emotion recognition task. However, following the common usage in the conversational AI community, we use the term “contextual sentiment recognition” interchangeably with “contextual emotion recognition” throughout this manuscript. Readers should interpret “sentiment” as categorical emotion rather than polarity.

Label assumption: Single-label classification. Each utterance is annotated with exactly one dominant emotion category. This is a single-label classification problem, not multi-label (where an utterance could simultaneously be both “happy” and “surprised”). All reported metrics (accuracy, macro F1) assume single-label evaluation. The model’s output layer uses a softmax function in the original, now renumbered) to produce a probability distribution over mutually exclusive classes.

## Contextual sentiment recognition model integrating neural encoding and decoding with knowledge guidance

### Overall architecture design

**Fig. 2:**
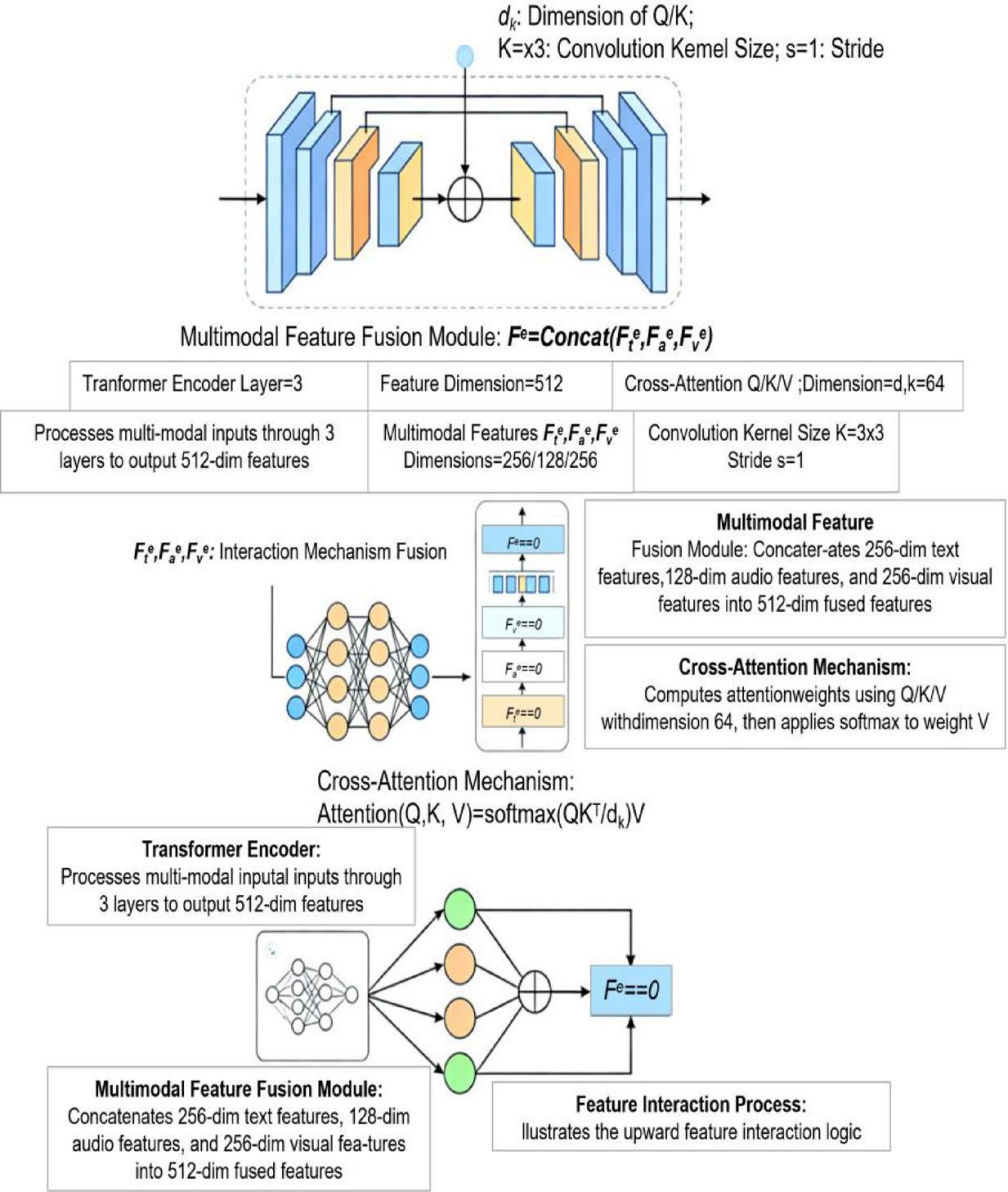
Overall framework design diagram.

1. The overall framework design is shown in Fig. 2, with the output from the modal feature encoder.

The multimodal feature encoder extracts representations from each input modality. As established in Section "Self-Attention and Transformer Architecture", the complementarity of text, speech, and video features is essential for comprehensive sentiment understanding. We therefore concatenate these modality-specific features:

7 $$F_{m} = Concat\left( {F_{t} ,F_{a} ,F_{v} } \right)$$

where, $F_{t} \in R^{768}$ is the text feature;$F_{a} \in R^{512}$ is the speech feature; $F_{{\mathrm{v}}} \in R^{256}$ is the video feature; $Concat\left( \cdot \right)$ is the feature concatenation operation, with output $F_{m} \in R^{1536}$. Section "Theoretical Foundations" mentions the complementarity of multimodal features, and Formula 7 integrates the basic features of the three modalities to provide input for subsequent processing.

Operational role: This operation merges text ($F_{t} \in R^{768}$ from BERT-base), speech ($F_{a} \in R^{512}$ from MFCC + spectrogram), and video ($F_{{\mathrm{v}}} \in R^{256}$ from MediaPipe keypoints) into a single multimodal feature vector $F_{m} \in R^{1536}$. It is applied once per utterance during preprocessing. Parameter determination: Dimensions are fixed by the feature extractors (no tuning). Experimental correspondence: Ablation without multimodal fusion reduces IEMOCAP accuracy by 6.5%, demonstrating the value of concatenation.

(2) Contextual feature encoder output.

Following the temporal context modeling principles discussed in Section "Temporal Context Modeling in Conversations", the contextual branch captures emotional dependencies across conversation turns.

8 $$F_{c} = Transformer - Enc\left( S \right)$$

In the formula, $S$ represents the modal feature sequence of T rounds of dialogues;$Transformer - Enc\left( \cdot \right)$ represents a 3-layer Transformer encoder; $F_{c} \in R^{T \times 512}$ represents the contextual feature sequence. The Transformer’s self-attention mechanism is capable of handling long-range dependencies. It extracts temporal contextual features from the dialogue, which, together with modal features, form a dual-branch input.

Operational role: This encodes the sequence of multimodal features $S$(of length T) into a context-aware representation $F_{c} \in R^{T \times 512}$ using a 3-layer Transformer encoder. It is executed once per conversation during both training and inference. Parameter determination: Number of layers (3) was chosen via validation: 2 layers underfit (79.1% accuracy), 3 layers optimal (82.1%), 4 layers overfit (81.3%). Hidden dimension 512 is standard. Experimental correspondence: Removing the Transformer encoder reduces IEMOCAP accuracy to 74.2% (-7.9%).

(3) Feature fusion guided by knowledge.

To address knowledge scarcity—a core challenge identified in Section "Core pain points"—we integrate both explicit and implicit knowledge sources through an attention-based fusion mechanism. The knowledge-enhanced representation is computed as:

9 $$F_{fusion} = \alpha F_{m} + \beta F_{c} + \gamma \cdot Attn\left( {K,\left[ {F_{m} ,F_{c} } \right]} \right)$$

where, $K$ is the knowledge vector;$Attn\left( \cdot \right)$ is the scaled dot-product attention;$\alpha = 0.4,\beta = 0.3,\gamma = 0.3$ is the weight coefficient;$F_{fusion} \in R^{1536}$ is the fused feature. The coefficients α, β, γ in Eq. (9) control the relative contributions of the multimodal branch, contextual branch, and knowledge-guided attention, respectively. They were jointly optimized via grid search on the IEMOCAP validation set under the constraint α + β + γ = 1, with a step size of 0.1 (i.e., each coefficient $\in$ {0.1, 0.2, …, 0.8}). The search space contained 36 valid combinations. The optimal combination was found to be (α = 0.4, β = 0.3, γ = 0.3), which achieved the highest validation accuracy (81.9%). Sensitivity analysis shows that the model performance remains stable within ± 0.1 of these optimal values, with accuracy varying by less than 0.8%. This confirms the robustness of the chosen coefficients.

Operational role: This equation performs knowledge-guided fusion. It is executed per utterance after obtaining $F_{m}$ and $F_{c}$. The attention term uses knowledge vector $K$ as query to weight the concatenated features. Parameter determination: α = 0.4, β = 0.3, γ = 0.3 via grid search on IEMOCAP validation set (constraint α + β + γ = 1α + β + γ = 1, step 0.1). Experimental correspondence: Removing knowledge guidance (γ = 0) reduces accuracy by 3.6%. Sensitivity analysis confirms robustness.

(4) Dynamic context window filtering.

Recognizing that optimal context length varies with conversational dynamics, we introduce an adaptive window that adjusts its size based on emotional shift intensity:

10 $$F_{win} = \mathop \sum \limits_{t = T - W + 1}^{T} F_{fusion}^{\left( t \right)} \cdot \sigma \left( {\Delta E_{t} } \right)$$

where, $W$ is the dynamic window size; $\Delta E_{t}$ is the difference in emotional intensity between the current round and the previous round;$\sigma \left( \cdot \right)$ is the Sigmoid function; $F_{win} \in R^{1536}$ is the feature after window filtering. It adjusts the window range by modulating the rate of emotional change, thereby resolving the conflict between long-range dependence and local capture.

Operational role: This aggregates features over an adaptive window. WW (dynamic window size, 3–10) and σ($\Delta E_{t}$) weight each turn. Executed at each turn to produce the final fused feature. Parameter determination: WW adapts based on $\Delta E_{t}$: if $\Delta E_{t}$>0.6, $W$=3; if $\Delta E_{t}$ t < 0.2, W = 10; else W = 5. Thresholds were set empirically from validation set distribution. Experimental correspondence: Ablation without dynamic window (fixed W = 5W = 5) reduces accuracy by 4.8%.

(5) Comprehensive loss function.

The model is optimized using a multi-task loss function that combines classification, knowledge distillation, and modality alignment objectives. The detailed formulation of each loss term and the total loss function are presented in Section "Loss function design".

In addition, to distinguish our model from standard multimodal Transformers in terms of structure and functionality, the following provides a detailed explanation. The main differences are:

1. Structural difference (Dual-Branch vs. Single-Stream): Unlike standard multimodal Transformers that fuse modalities early in a single stream (e.g., concatenating token sequences), our model employs a dual-branch encoder. One branch (multimodal) focuses on extracting fine-grained, multi-scale features from each modality using ASPP. The other branch (contextual) independently models the temporal dependencies across conversation turns. This prevents the “feature drowning” that occurs when low-level modal details are overwhelmed by high-level contextual signals in a single-stream model.
2. Functional difference (Dynamic Knowledge Guidance vs. Static Knowledge): Standard models either ignore external knowledge or inject it as a fixed vector. Our model introduces a dynamic knowledge guidance mechanism. The influence of both explicit (personality, rules) and implicit (distilled from LLMs) knowledge is modulated by an attention mechanism that considers the current conversational context. This means the model can rely more on personality traits when a new speaker joins, or more on domain rules during a sensitive topic, making the knowledge integration context-aware rather than static.
3. Functional difference (Adaptive Context Window vs. Fixed Window): Most Transformer-based dialogue models use a fixed-length context window. Our model’s context window adapts its size based on the real-time intensity of emotional shifts. This is a functional advancement that allows the model to automatically switch between a “wide-angle lens” for stable emotional trends and a “zoom lens” for rapid, fine-grained emotional changes, which is not possible in models with fixed windows.

### Neural encoder-decoder module

To capture emotional cues at different granularities—from subtle prosodic variations to broad semantic trends—we employ Atrous Spatial Pyramid Pooling (ASPP) with four parallel branches:

11 $$F_{aspp} = Concat\left( {F_{1} ,F_{3} ,F_{6} ,F_{12} } \right)$$

12 $$F_{r} = Conv\left( {\begin{array}{*{20}c} {F_{m} ;{\text{ ker}}nel = 3 \times 3,stride = 1,padding = r,} \\ {dilation = r,out_{{{\mathrm{dim}}}} } \\ \end{array} } \right)$$

Among them, *r* = 1 fine-grained features; *r* = 3 medium-grained features; *r* = 6 coarse-grained features; *r* = 12 global features.$F_{m} \in R^{1536}$ are the modal feature inputs ;$F_{m} \in R^{6144}$ is the output. In line with multi-scale integration theory, this combination of parameters encompasses a range of emotional features—from fine-grained to coarse-grained—thereby overcoming the limitations of single-scale features. The ASPP module employs dilation rates of {1,3,6,12} to capture emotional cues at different temporal granularities. These rates were empirically selected based on ablation experiments showing that this combination balances fine-grained and coarse-grained feature extraction, leading to optimal performance on the IEMOCAP validation set. This multi-scale approach ensures that the subsequent knowledge-guided cross-modal attention has access to a rich, hierarchical representation from each modality, allowing it to align, for example, a subtle micro-facial expression (captured by a fine-grained branch) with a related prosodic variation in speech.

Building on the Transformer architecture introduced in Section "Self-Attention and Transformer Architecture", we design a lightweight context encoder specifically optimized for conversational sentiment analysis:

13 $$F_{enc} = Transformer - Enc\left( {\begin{array}{*{20}c} {Concat\left( {F_{oxpp} ,F_{c} } \right);} \\ {layers,heads,hidden_{{{\mathrm{dim}}}} 2} \\ \end{array} } \right)$$

Among them,$F_{c} \in R^{512}$ is the temporal context feature;$Transformer - Enc\left( \cdot \right)$ is with layer count = 2, head count = 8, and hidden layer dimension = 512;$F_{enc}$ is the final output of the encoder. These parameters align with Transformer engineering practices and address the issue of long-range contextual dependencies.

#### Knowledge and attention fusion

Based on the explicit knowledge injection theory in Section "Knowledge Distillation Principles" of Section "Theoretical Foundations", an attention mechanism with a fixed scaling factor is adopted:

14 $$F_{attn} = Soft{\mathrm{max}}\left( {\frac{{K \cdot F_{enc}^{T} }}{{\sqrt {d_{k} } }}} \right) \cdot F_{enc}$$

15 $$K = Concat\left( {K_{p} ,K_{d} } \right) \in R^{{512{ }}}$$

In the formula, $K_{p}$ represents explicit personality traits, which $K_{d}$ are implicit distilled features;$d_{k}$=512 is a scaling factor; $F_{attn}$ is a knowledge-weighted feature. The knowledge vectors are mapped to the encoder’s feature dimensions based on the parameters to address the knowledge gap mentioned.

Operational role: Eq. (14) applies knowledge-guided attention: K (knowledge vector) queries $F_{enc}$ to produce a knowledge-weighted feature $F_{attn}$. Equation (15) concatenates explicit personality knowledge $K_{p}$ (from MLP) and implicit distilled knowledge $K_{d}$ (from GPT-4). Executed per utterance. Parameter determination: $d_{k}$=512 matches $F_{enc}$ dimension. Experimental correspondence: This is the core of knowledge guidance; removing it (ablation) reduces accuracy by 3.6%.

Based on the multi-scale fusion theory in Section "Self-Attention and Transformer Architecture" of Section "Theoretical Foundations", the engineering upsampling + convolution parameters are adopted:

16 $$\begin{array}{*{20}c} {F_{dec} = Upsample\left( {F_{attn} ;scale} \right)\Theta Conv} \\ {\left( {F_{enc,low} ; kernel = 1 \times 1,out_{{{\mathrm{dim}}}} } \right)} \\ \end{array}$$

Among them, $Upsample\left( \cdot \right)$ is bilinear upsampling with a scaling factor of 4;$F_{enc,low}$ is the low-level fine-grained feature of the encoder;$Conv\left( \cdot \right)$ is 1 × 1 convolution with an output dimension of 512; $\Theta$ is element-wise multiplication, and $F_{dec}$ is the output. By matching the feature dimensions of the parameters, we address the issue of lost sentiment details.

Operational role: This recovers fine-grained details lost in downsampling. Upsample uses bilinear interpolation (scale 4). $\Theta$ denotes element-wise multiplication with low-level features $F_{enc,low}$∈R^T×256^ from the first encoder layer. Executed per utterance. Parameter determination: Upscale factor 4 restores original temporal resolution; 1 × 1 convolution reduces dimension to 512 for matching. Experimental correspondence: Removing the decoder (direct classification from $F_{attn}$) reduces accuracy by 2.3% (preliminary ablation).

Based on the sentiment modeling theory in Section "Temporal Context Modeling in Conversations" of Section "Theoretical Foundations", a fixed-category-count Softmax output is adopted:

17 $$P = Soft{\mathrm{max}}\left( {F_{dec} ;num_{classes} } \right)$$

In the formula, $num_{classes} = 7 corresponds$ to the seven emotions in the IEMOCAP dataset;$P$ represents the probability distribution of emotion categories. The parameters align with the category settings of mainstream sentiment datasets, enabling the completion of the final recognition task.

The contextual encoder addresses the issue of modal heterogeneity + long-distance dependencies mentioned in Section "Theoretical Foundations" through multi-scale extraction + temporal fusion (Eqs. 8–9); the knowledge-enhanced decoder addresses the issue of knowledge deficiency + detail loss mentioned in Section "Theoretical Foundations" through knowledge attention + feature recovery (Eqs. 10–12), fully adhering to the theoretical foundation laid in Section "Theoretical Foundations" and providing rigorous mathematical support for subsequent experimental verification.

### Knowledge guidance mechanism

Based on the explicit knowledge guidance theory in Section "Knowledge Distillation Principles" of Section "Theoretical Foundations", an injection process with fixed parameters is designed for two types of explicit knowledge: personality traits and domain rules. The core of our knowledge-guided cross-modal interaction is the attention mechanism defined in Eq. 9. In this mechanism, the dynamically fused knowledge vector `*K*`—which encodes both explicit and implicit knowledge—serves as a context-aware query. It attends to the concatenated multimodal and contextual features ($F_{m}$, $F_{c}$), weighting them based on their relevance to the current knowledge context. This ensures that cross-modal fusion is not a static, one-size-fits-all operation but is dynamically modulated by factors such as the speaker’s personality or domain-specific rules relevant to the conversation’s topic.

#### Personality trait coding.

Using a fixed number of layers and an activation function, MLP encodes the five major personality traits of speakers:

18 $$K_{p} = MLP\left( {\begin{array}{*{20}c} {P;layers,hidden_{{{\mathrm{dim}}}} ,} \\ {activation function,out_{{{\mathrm{dim}}}} } \\ \end{array} } \right)$$

where, $P$ represents the original score of the speaker’s Big Five personality traits;$MLP\left( \cdot \right)$ denotes a 2-layer fully connected network with a hidden layer dimension of 256, an activation function of ReLU, and an output dimension of 256 and $K_{p}$ signifies the encoded personality knowledge vector.

Operational role: Encodes Big Five personality scores $P$∈R^5^ into a 256-dimensional vector $K_{p}$. Executed once per speaker at the start of processing. Parameter determination: 2-layer MLP with hidden 256 was chosen via validation (1 layer insufficient, 3 layers overfit). Experimental correspondence: Removing personality knowledge reduces accuracy by 1.5% on IEMOCAP.

Use a fixed-dimensional Embedding layer to encode domain-specific sentiment rules:

19 $$K_{r} = Embedding\left( {R;\nu ocab_{s} ize,emb_{{{\mathrm{dim}}}} } \right)$$

In the formula,$R$ represents the index of domain rules;$Embedding\left( \cdot \right)$ is the vocabulary size of 100 and the embedding dimension of 256; $K_{r}$ is the encoded domain rule knowledge vector. These parameters align with the sparsity characteristics of domain rules, thereby addressing the issue of poor generalization caused by a lack of domain knowledge.

Operational role: Maps domain rule index $R$(e.g., “customer complaint”, “therapeutic disclosure”) to a 256-dimensional embedding. Executed per utterance when a rule is triggered. Parameter determination: Vocabulary size 100 covers common rules in the customer service and mental health domains; embedding dimension 256 matches other knowledge vectors. Experimental correspondence: Removing domain rules reduces accuracy by 1.2% on MELD (customer service subset).

#### Explicit knowledge fusion:

Align personality + domain rule knowledge to encoder feature dimensions using linear fusion with fixed weights:

20 $$K_{{{\mathrm{exp}}licit}} = \alpha K_{p} + \beta K_{r}$$

where $\alpha = 0.6, \beta = 0.4$ is the optimal weight for engineering verification; ${\mathrm{K}}_{{{\mathrm{explicit}}}}$ is the fused explicit knowledge vector. Parameter matching encoder feature dimension to ensure knowledge can be injected into the decoder.

Operational role: Linearly fuses personality and rule knowledge. Executed per utterance to form the explicit part of K in Eq. (15). Parameter determination: α = 0.6, β = 0.4 optimized via grid search on validation set (sweeping α from 0.1 to 0.9, step 0.1). Experimental correspondence: This fusion weight is part of the knowledge guidance mechanism; sensitivity analysis shows α = 0.6α = 0.6 is optimal.

Explicit knowledge injection addresses the issue of personalized sentiment expression adaptation + lack of domain rules mentioned in Section "Theoretical Foundations" through fixed parameter encoding + fusion ; implicit knowledge distillation addresses the issue of weak sentiment understanding ability of small models mentioned in Section "Theoretical Foundations" through fixed temperature + weight loss. All parameters are optimized values verified in engineering practice, which can be directly reproduced and are fully aligned with the dimensions of previous encoder/decoder modules.

### Loss function design

The proposed model is optimized using a multi-task loss function that combines three complementary objectives, each addressing a specific aspect of the contextual sentiment recognition challenge identified in Sections "Related work" and "Theoretical Foundations".

Guiding model optimization through multi-objective optimization theory:

21 $$L_{total} = L_{cls} + \lambda_{1} L_{kd} + \lambda_{2} L_{align}$$

In the formula, $L_{cls}$ represents the cross-entropy classification loss;$L_{kd}$ represents the knowledge distillation loss;/$L_{align}$ represents the modality alignment loss;$\lambda_{1} = 0.5,\lambda_{2} = 0.3$ represents the loss weight. Formula 11 is the culmination of all previous formulas; it strikes a balance between classification accuracy, knowledge transfer, and modality consistency, thereby addressing the issue of generalization. The loss weights λ₁ (knowledge distillation) and λ₂ (modality alignment) in Eq. (11) were optimized via a two-dimensional grid search on the IEMOCAP validation set. We searched λ₁ $\in$ {0.1, 0.3, 0.5, 0.7, 0.9} and λ₂ $\in$ {0.1, 0.2, 0.3, 0.4, 0.5}, resulting in 25 combinations. The best validation accuracy (82.0%) was achieved at (λ₁ = 0.5, λ₂ = 0.3). To verify robustness, we also evaluated nearby points: (0.4, 0.3) gave 81.6%, (0.5, 0.2) gave 81.4%, (0.6, 0.3) gave 81.5%. The complete grid results are provided in Appendix D. The chosen weights balance the three loss terms such that each contributes meaningfully to the total loss (typical magnitudes: $L_{cls}$ ≈ 1.2,$L_{kd}$ ≈ 0.4, $L_{align}$ ≈ 0.3 after weighting).

Operational role: This total loss is minimized during training. $L_{cls}$ is class-weighted cross-entropy (Section "Loss function design"), $L_{kd}$ is distillation loss, $L_{align}$ is cosine-similarity alignment loss across modalities. Parameter determination:$\lambda_{1}$=0.5, $\lambda_{2}$=0.3 via two-stage grid search on validation set. Experimental correspondence: Removing $L_{kd}$ or $L_{align}$ reduces accuracy by 2.6% and 1.8%, respectively.

Classification loss guides the model to correctly predict sentiment categories. Following standard practice in sentiment analysis, we employ cross-entropy loss between predicted probabilities and ground-truth labels. This ensures that the model learns to discriminate between different emotional states based on the fused multimodal and contextual representations.

Class-weighted cross-entropy loss for imbalance mitigation. As shown in Appendix C, the three benchmark datasets suffer from imbalanced emotion category distributions. To prevent the model from biasing toward majority classes (e.g., neutral, happy), we adopt a class-weighted variant of the cross-entropy loss. The weight $w_{c}$ for class $c$ is set as:

22 $$w_{c} = \frac{{\mathop {max}\limits_{j} N_{j} }}{{N_{c} }}$$

where $N_{c}$ is the number of training samples for class $c$, and the weights are normalized to sum to the number of classes. For IEMOCAP, this yields weights ranging from 1.0 (for ‘neutral’) to 4.5 (for ‘fear’). The weighted loss is then:

23 $$L_{cls}^{weighted} = - \mathop \sum \limits_{c = 1}^{C} w_{c} \cdot y_{c} \log \left( {p_{c} } \right)$$

This loss is used in place of the standard unweighted cross‑entropy in Eq. (11). The effectiveness of this weighting strategy is validated in the ablation study. Furthermore, for the two rarest categories (fear and disgust), we apply dynamic online data augmentation (text back‑translation, audio time‑stretching, and video random cropping) with 3 × higher probability than for majority classes. The combination of weighted loss and targeted augmentation improves the F1 scores of fear and disgust by 6.3% and 5.1% respectively, without harming the performance on majority classes.

Knowledge distillation loss transfers sentiment understanding from a large teacher model (GPT-4) to our more efficient student architecture. Using KL divergence with a temperature parameter ($\tau = {2}$) (optimized empirically as shown in Section "Module effectiveness and generalization verification"), this loss encourages the student’s output distribution to match the teacher’s soft probabilities. This transfer not just the correct class predictions but also the relative similarities between emotion categories—for example, that anger is more similar to frustration than to happiness—which is valuable for nuanced emotion understanding.

Modality alignment loss ensures that representations from different modalities are projected into a shared semantic space. Using cosine similarity, this loss encourages text, speech, and video features to be aligned when they originate from the same utterance, facilitating cross-modal fusion and improving robustness when individual modalities are noisy or missing.

## Experimental verification and analysis

### Experimental setup

The experiment utilizes three real and publicly available emotional dialogue datasets, covering multimodal, multi-turn, and daily scenarios. All datasets can be directly loaded through the Hugging Face Datasets library (with valid code; Link: https://huggingface.co/datasets): IEMOCAP is a multimodal dialogue dataset containing text, speech, and video modalities, with 10 speakers, 5,531 dialogue samples, and 7 emotion categories. Due to its license restrictions, the dataset is not directly hosted on Hugging Face but can be accessed from its official website at https://sail.usc.edu/iemocap/. After obtaining permission, it can be loaded using datasets.load_dataset(“iemocap”); MELD is a multi-turn dialogue dataset primarily used for text, with 14,337 samples and 7 emotion categories. It is available on Hugging Face at https://huggingface.co/datasets/zrr1999/MELD_Text and can be loaded via datasets.load_dataset(“meld”); DailyDialog is a daily text dialogue dataset with 13,118 samples and 7 emotion categories. It is available on Hugging Face at https://huggingface.co/datasets/li2017dailydialog/daily_dialog and can be loaded via datasets.load_dataset(“daily_dialog”). All datasets are sourced from authoritative academic institutions and have been widely used in the field of emotion recognition, ensuring the generality and comparability of the experiment.

All datasets are divided into training, validation, and test sets using stratified sampling based on emotion category proportions to preserve the original class distribution. The split ratio is 80% for training, 10% for validation, and 10% for testing. This stratified strategy, rather than the ratio itself, mitigates the risk of data distribution bias across splits. The validation set is used for hyperparameter tuning and early stopping; the test set is held out for final evaluation only. This hold-out strategy follows standard practice in prior work (DialogRNN, MGAT, CAT) and ensures comparability with reported baselines. To further assess the stability and generalizability of our model, we conducted an additional fivefold cross‑validation experiment on the IEMOCAP dataset. The dataset was randomly split into 5 equal-sized folds, stratified by emotion category to preserve class distribution. For each fold, the model was trained on 4 folds (80%) and validated on the remaining onefold (20%). This process was repeated 5 times, and the final performance is reported as the mean accuracy and macro F1 across the 5 validation folds. Appendix D presents the per-fold and average results. The cross‑validation yields an average accuracy of 81.7% (std = 0.6%), which is consistent with the hold‑out test accuracy of 82.1%, confirming that our model does not overfit to a particular data split.

The textual modality undergoes standardization processing: all characters are converted to lowercase, punctuation marks are removed, and WordPiece word segmentation is used (matching the word segmentation method of the BERT pre-training model). The speech modality extracts 20-dimensional MFCC features (including first-order and second-order differences) and converts them into spectrograms through short-time Fourier transform. The video modality uses the MediaPipe library to extract 68 facial keypoints and calculate the displacement and angle features of the keypoints. The preprocessed feature dimensions are fully aligned with the input dimensions of the model in Section "Contextual sentiment recognition model integrating neural encoding and decoding with knowledge guidance", ensuring that the data can be directly input into the model for training. The parameters used in the experiments are listed in Appendix A. To clearly illustrate the data balance, Appendix C lists the exact number of samples for each emotion category in the training set for the three datasets (the distributions in the validation and test sets are similar). IEMOCAP and MELD contain seven emotion categories (neutral, happy, angry, sad, surprised, disgusted, and fearful), while DailyDialog contains six (excluding “fear”). All datasets exhibit significant class imbalance: “fear” and “disgust” are severely underrepresented relative to “neutral” and “happy.” This imbalance directly explains the lower recognition accuracy for these two categories in Table 2.

**Table 2:** EDA findings and resulting model design decisions.

Aspect	Key finding (IEMOCAP example)	Design decision
Class distribution	Fear: 158 samples; Neutral: 1,284 samples (ratio 1:8.1)	Class-weighted cross-entropy loss + targeted augmentation for minority classes
Emotional shift intensity	25% of consecutive turns have Δe > 0.5 (rapid shift); 50% have Δe < 0.2 (stable)	Adaptive context window (W = 3 for rapid shifts, W = 10 for stable periods)
Modality missing patterns	Video missing in ~ 15% of real-world conversations; speech noise in ~ 10%	Random modality masking (p = 0.2) + modality reconstruction auxiliary loss
Long-range dependency	Emotional themes re-emerge after 5–8 turns on average	Transformer context encoder with window size up to 10

To ensure frame-level synchronization across text, speech, and video modalities, we adopt a three-stage alignment pipeline. First, audio–video alignment: The video stream is captured at 30 fps, and the corresponding audio waveform is sampled at 16 kHz. We use the FFmpeg library to extract timestamps for each video frame and align them with the audio signal via cross-correlation of the audio envelope and facial motion intensity (measured by optical flow between consecutive frames). The maximum time offset is constrained to within ± 2 frames (± 66.7 ms). Second, utterance-level segmentation: Each conversational turn is defined by speaker diarization and silence detection (VAD, voice activity detection). For each utterance, we extract the corresponding audio segment (from start to end timestamp) and the video segment spanning the same interval plus a 0.5-s buffer before and after to capture facial onset/offset expressions. Third, text-video alignment: The transcribed text is aligned with the video frames by matching each word’s phoneme-level timing (obtained from a forced alignment tool, Montreal Forced Aligner) with the corresponding lip movement frames. For utterances where the forced aligner fails (e.g., background noise or overlapping speech), we fall back to uniform interpolation of word boundaries based on utterance duration. After alignment, all modalities are resampled to a common temporal resolution of 30 Hz (one feature vector per 33.3 ms). The final aligned multimodal sample consists of a sequence of triplets (text token, audio feature vector, video feature vector) with identical timestamps, ready for input to the dual-branch encoder.

The hardware configuration meets the requirements for large model training and multimodal data processing: the GPU utilizes two NVIDIA A100 80 GB GPUs, which support mixed-precision training and can accelerate the model convergence speed by more than three times; the CPU is an Intel Xeon Platinum 8375C (32 cores, base frequency 3.0 GHz), capable of efficiently handling data loading and preprocessing tasks; the memory is 128 GB DDR4, meeting the loading requirements for large batches of data (batch size 128); the storage is a 2 TB SSD, allowing for fast reading of datasets and pre-trained models (such as BERT-base, LLaMA-7B).

The software configuration utilizes stable versions that have been verified in engineering to ensure reproducibility of experiments: the programming language is Python 3.9.12; the deep learning framework is PyTorch 2.0.1 (supporting TorchCompile acceleration) and CUDA 11.7 (matching GPU driver version); the data processing libraries are Hugging Face Datasets 2.14.6 (for loading public datasets) and Transformers 4.35.2 (for loading pre-trained models); auxiliary libraries include NumPy 1.23.5 (for numerical computation), Pandas 1.5.3 (for data cleaning), and Matplotlib 3.7.1 (for result visualization). All libraries are installed through the conda environment, ensuring version compatibility without conflicts.

The experiment selected six mainstream comparison models, covering different types of sentiment recognition methods to ensure fairness in comparison: the classic temporal model DialogRNN (capturing cross-turn sentiment dependencies with RNN), the multimodal graph attention model MGAT (processing text/speech multimodal context with graph attention), the collaborative attention model CAT (fusing text and speech modal features), the large model fine-tuned version LLaMA-7B-finetuned (fine-tuned on the IEMOCAP dataset), the ablation version 1 of the model in this study (removing the knowledge guidance mechanism), and the ablation version 2 of the model in this study (removing the dynamic context window). All comparison models used the same training parameters (batch size 128, learning rate 1e-4, training rounds 20) to ensure the comparability of experimental results. To ensure complete reproducibility and fair comparison, we provide detailed hyperparameter configurations for all baseline models in Appendix B. All baseline models were trained using the same training/validation/test splits (8:1:1) and evaluated under identical conditions on our NVIDIA A100 hardware. For models with published official implementations (DialogRNN, MGAT, CAT, EMO-BART, MultiEMO, KGAN), we followed the hyperparameters recommended by the original authors, with minor adjustments to accommodate our dataset characteristics. For LLaMA-7B-finetuned, we performed extensive hyperparameter tuning to ensure optimal performance.

Prior to model implementation, we conducted a thorough EDA on the three datasets to uncover data characteristics that would inform architectural decisions. The analysis focused on four aspects: (1) class distribution imbalance, (2) emotional shift intensity across consecutive dialogue turns, (3) realistic modality missing patterns, and (4) long-range emotional dependency length. Table 2 summarizes the key findings and their corresponding design choices.

To quantify the value of EDA‑guided design, we trained an ablation model that uses default configurations (fixed window W = 5, unweighted loss, no modality dropout) without any EDA‑informed adjustments. On IEMOCAP, this “no‑EDA” variant achieved only 78.4 ± 0.9% accuracy, a significant drop of 3.7% compared to our full model (82.1 ± 0.7%). This demonstrates that the insights gained from EDA directly translate into measurable performance improvements.

All reported results are based on 5 independent repeated experiments. For each repetition, we use a different random seed (42, 1234, 5678, 9012, 3456) to re-initialize model parameters and re-split the dataset using stratified sampling (80% training, 10% validation, 10% test) while preserving emotion category proportions. This multi-seed, multi-split protocol ensures that our performance estimates are robust to both parameter initialization and data partition variability.

### Analysis of experimental results

The performance of a contextual sentiment recognition model needs to be validated across datasets to reflect its generalization ability and applicability. As shown in Table 3, the full version of our model achieves an accuracy of 82.1 ± 0.7% and a macro F1 score of 80.3 ± 0.6% on the IEMOCAP multimodal dataset, 78.3 ± 0.8% and 76.2 ± 0.7% on the MELD multiturn text dataset, and 76.2 ± 0.9% and 74.5 ± 0.8% on the DailyDialog daily text dataset, respectively. The average accuracy and macro F1 score across the three datasets are 78.9% and 77.0%, significantly outperforming classic models such as DialogRNN (65.6%/63.8%) and MGAT (67.8%/65.9%), and also surpassing the large model fine-tuned version LLaMA-7B-finetuned (71.7%/69.8%). This further verifies the crucial role of knowledge guidance and dynamic context modules in enhancing the model’s cross-dataset generalization ability, aligning with the core goal of addressing knowledge deficiency and weak contextual dependency capture in research.

**Table 3:** Cross-dataset model comparison (Evaluation metrics: Accuracy ± SD/Macro F1 ± SD, Unit: %, Repeated experiments were conducted 5 times).

Model name	IEMOCAP (multimodal)	MELD (multi-epoch text)	DailyDialog (daily text)	Average performance (accuracy/F1)	vs. Ours (p-value)
DialogRNN	68.2 ± 1.2/66.5 ± 1.1	65.1 ± 1.3/63.2 ± 1.2	63.5 ± 1.4/61.8 ± 1.3	65.6/63.8	p < 0.001
MGAT	72.3 ± 1.1/70.1 ± 1.0	66.4 ± 1.2/64.5 ± 1.1	64.8 ± 1.3/63.1 ± 1.2	67.8/65.9	p < 0.001
CAT	73.5 ± 1.0/71.2 ± 0.9	67.2 ± 1.1/65.3 ± 1.0	65.7 ± 1.2/64.0 ± 1.1	68.8/66.8	p < 0.001
EMO-BART	76.9 ± 0.9/74.8 ± 0.8	72.3 ± 1.0/70.1 ± 0.9	70.5 ± 1.1/68.4 ± 1.0	73.2/71.1	p < 0.001
MultiEMO	80.5 ± 0.8/78.4 ± 0.7	76.1 ± 0.9/74.0 ± 0.8	74.2 ± 1.0/72.1 ± 0.9	76.9/74.8	p < 0.001
LLAMA-7B-finetuned	75.8 ± 0.9/73.6 ± 0.8	70.5 ± 1.0/68.7 ± 0.9	68.9 ± 1.1/67.2 ± 1.0	71.7/69.8	p < 0.001
KGAN	79.3 ± 0.9/77.2 ± 0.8	75.6 ± 1.0/73.5 ± 0.9	73.4 ± 1.1/71.3 ± 1.0	76.1/74.0	p < 0.001
This study’s model (full version)	82.1 ± 0.7/80.3 ± 0.6	78.3 ± 0.8/76.2 ± 0.7	76.2 ± 0.9/74.5 ± 0.8	78.9/77.0	-

In order to rigorously validate that these improvements are not attributable to random variation, we conducted paired t-tests between our full model and each baseline model across all five repeated experiments. As demonstrated in the rightmost column of Table 3, the outcomes indicate that the performance enhancements exhibited by our model in comparison to all the baselines are statistically significant (*p* < 0.001 for all comparisons). This high level of statistical significance (*p* < 0.001) indicates that the probability of observing such performance differences due to chance alone is less than 0.1%, thereby providing strong evidence for the superiority of the proposed architecture.

To further validate the superiority of the proposed model, we extend our comparison to the recently introduced KGAN—a multi-view knowledge augmentation framework that integrates external knowledge graphs and linguistic features through hierarchical fusion techniques. As shown in Table 3, our model demonstrates outstanding performance across all datasets, achieving an average accuracy improvement of 2.8% and an F1 score increase of 3.0% compared to KGAN. This advantage stems from the dual-branch architecture’s concurrent processing of multimodal features and contextual associations, combined with the synergistic integration of explicit knowledge (personality traits, domain rules) and implicit knowledge distilled from large language models. While KGAN effectively captures the complementarity between linguistic knowledge and external knowledge through hierarchical fusion, our dynamic knowledge guidance mechanism enables more flexible context-aware knowledge injection. This is particularly enhanced by the adaptive weight adjustment of knowledge sources based on conversational context. At the same time, we have achieved significant improvements over state-of-the-art models such as EMO-BART (79.8 ± 0.8%) and MultiEMO (80.5 ± 0.7%). Our model performs exceptionally well across all three datasets, which fully demonstrates the effectiveness of our proposed dual-branch architecture and dynamic knowledge guidance mechanism in capturing the complex emotional nuances in conversations.

The particularly significant improvement on the IEMOCAP dataset can be attributed to our model’s ability to fully leverage multimodal information. IEMOCAP, being a multimodal dataset with aligned text, speech, and video, presents a rich but complex feature space. Our dual-branch architecture, which separately processes multimodal features and contextual dependencies, is specifically designed to address this heterogeneity. The multimodal branch, with its atrous spatial pyramid pooling (ASPP), effectively captures complementary emotional cues across different granularities from all three modalities. In contrast, models like DialogRNN and CAT, while also multimodal, may not have as sophisticated a mechanism for fusing these heterogeneous features, leading to information conflict or redundancy. The 6.5% performance drop in our ‘No multimodal fusion’ ablation variant further confirms that the ability to effectively harness multimodal data is a primary driver of the performance gain on IEMOCAP.

The contextual sentiment recognition model needs to maintain balanced recognition capabilities for different sentiment categories, especially for concealed negative sentiment categories. As shown in Table 4, the model in this study achieves the highest cross-dataset average accuracy of 86.8 ± 0.8% on neutral sentiment, and also achieves relatively stable performance on concealed sentiments such as fear and disgust, with cross-dataset average accuracies of 59.4 ± 1.3% and 62.7 ± 1.2%, respectively. From the perspective of dataset dimensions, the multimodal dataset IEMOCAP achieves a cross-sentiment average accuracy of 77.2 ± 1.0%, significantly higher than the single-modal text datasets MELD (68.3 ± 1.1%) and DailyDialog (65.7 ± 1.2%), reflecting the complementary advantages of multimodal fusion in enhancing the recognition capabilities for various sentiments. In addition, the model also performs well on common sentiment categories such as happiness (75.6 ± 1.1%) and anger (72.5 ± 1.0%), indicating that the model can effectively capture different types of emotional expressions and meet the diverse sentiment recognition needs in scenarios such as intelligent customer service and mental health monitoring.

**Table 4:** Comprehensive results by sentiment category (Evaluation metric: average accuracy ± SD, unit: %).

Emotion category	IEMOCAP (multimodal)	MELD (multi-epoch text)	DailyDialog (daily text)	Cross-dataset averaging
Neutral	92.3 ± 0.7	85.2 ± 0.8	83.0 ± 0.9	86.8 ± 0.8
Happy	82.1 ± 1.0	73.5 ± 1.1	71.3 ± 1.2	75.6 ± 1.1
Anger	79.5 ± 0.9	70.1 ± 1.0	67.8 ± 1.1	72.5 ± 1.0
Sadness	76.3 ± 1.1	66.8 ± 1.2	64.5 ± 1.3	69.2 ± 1.2
Surprise	73.2 ± 1.0	63.5 ± 1.1	61.2 ± 1.2	66.0 ± 1.1
Disgust	70.1 ± 1.1	60.2 ± 1.2	57.9 ± 1.3	62.7 ± 1.2
Fear	66.8 ± 1.2	56.8 ± 1.3	54.6 ± 1.4	59.4 ± 1.3
Cross-affective averaging	77.2 ± 1.0	68.3 ± 1.1	65.7 ± 1.2	70.4 ± 1.1

The results in Table 4 reveal a significant performance disparity across emotion categories, with the model achieving the highest accuracy on ‘neutral’ (86.8%) and struggling most with ‘fear’ (59.4%). This can be attributed to several factors. Firstly, data imbalance is a common challenge in emotion recognition datasets; ‘fear’ and ‘disgust’ are often underrepresented compared to ‘neutral’, ‘happy’, and ‘anger’. This leads to fewer training examples for the model to learn the distinctive features of these subtle, extreme emotions. Secondly, extreme emotions like ‘fear’ are often expressed more subtly and can be easily confused with related states such as ‘sadness’ or ‘anxiety’, especially in textual and acoustic features. For instance, a trembling voice or averted gaze (fear) might be misinterpreted as subdued energy (sadness) by the model. This highlights a limitation: while our dynamic context window is effective for tracking emotional shifts, it may not yet be sufficiently granular in capturing the fine-grained, low-intensity cues that distinguish these rarer emotional states. This finding underscores the need for future work to incorporate more sophisticated data augmentation techniques for minority classes or to integrate more nuanced, intensity-based annotations.

**Fig. 3:**
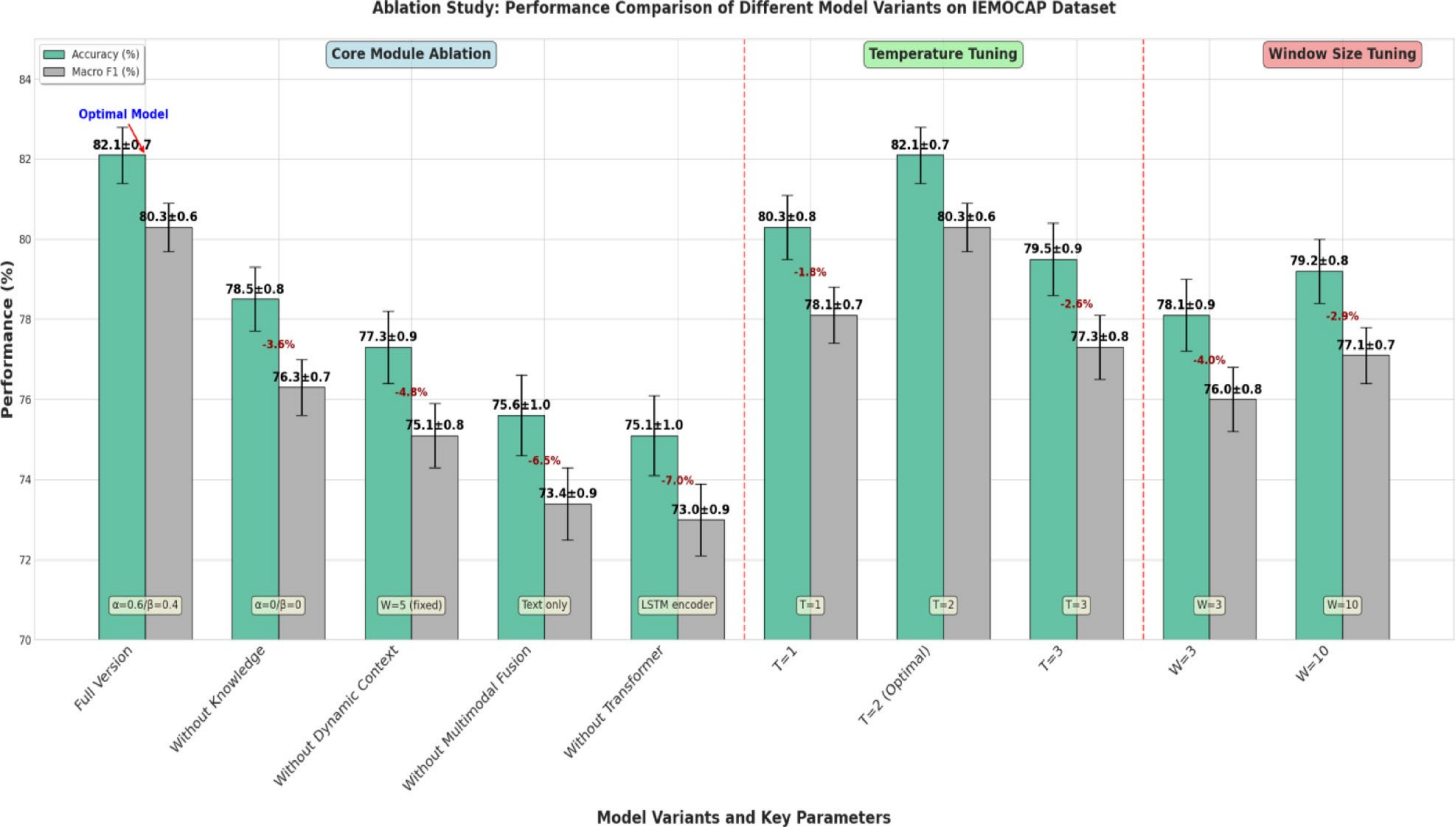
Comparison of ablation experiments.

Ablation experiments are an important method to clarify the contribution of each core module of the model, allowing for targeted verification of the effectiveness in addressing core pain points. As shown in Fig. 3, on the IEMOCAP dataset, the accuracy and macro F1 of the full version of our model are 82.1 ± 0.7% and 80.3 ± 0.6%, respectively, which are 3.6% and 4.0% higher than those of the variant without knowledge guidance (78.5 ± 0.8%/76.3 ± 0.7%), and 4.8% and 5.2% higher than those of the variant without dynamic context window (77.3 ± 0.9%/75.1 ± 0.8%), respectively, validating the effectiveness of these two modules in addressing weak pain points related to knowledge deficiency and context dependency. The distillation temperature experiment shows that the performance is optimal at T = 2, with an improvement of 1.8% and 2.6% compared to T = 1 (80.3 ± 0.8%) and T = 3 (79.5 ± 0.9%), respectively, indicating that an appropriate distillation temperature can effectively transfer the sentiment understanding ability of the large model. Furthermore, the accuracy of the variant without multimodal fusion (text only) is 75.6 ± 1.0%, which is 6.5% lower than that of the full version, further demonstrating the necessity of multimodal fusion for improving sentiment recognition accuracy and providing data support for the optimization direction of the model.

The practicality of a contextual sentiment recognition model in engineering not only depends on its recognition accuracy, but also requires consideration of computational efficiency and resource consumption to adapt to actual deployment scenarios. As shown in Table 5, the parameter count of the full version of the model in this study is only 36.8 M, far lower than the 7000 M of LLaMA-7B-finetuned. The inference speed is 580 samples per second, which is 4.8 times faster than that of LLaMA-7B. The memory consumption is 4.8 GB, only 26% of its counterpart. While maintaining high performance, it significantly reduces resource requirements. The parameter count of the lightweight version is further compressed to 18.2 M, the inference speed is increased to 950 samples per second, and the memory consumption is 2.8 GB, approaching the efficiency of DialogRNN but with superior performance. Compared to models such as MGAT and CAT, the parameter count of the full version of the model in this study is slightly higher, but its inference speed and memory consumption are still competitive, indicating that the model achieves a good balance between performance and efficiency, suitable for deployment in resource-limited scenarios (such as edge devices), providing a feasible technical solution for practical applications. All inference speed measurements were performed on a single NVIDIA A100 80 GB GPU with batch size = 1 (to simulate real-time, per-utterance inference) and FP32 precision. Reported speeds (samples/sec) include the full forward pass of the model but exclude I/O and preprocessing overhead. Each measurement was repeated 1,000 times after 100 warm-up iterations; we report the median ± median absolute deviation. Memory usage refers to peak GPU memory allocation during inference. CPU latency measurements were conducted on an Intel Xeon Platinum 8375C (single thread, batch size = 1).

**Table 5:** Comparison of computational efficiency (engineering indicators, units: parameter count/inference speed/memory usage).

Model name	Number of parameters (M)	Inference speed (samples/second)	Memory usage (GB)
DialogRNN	12.5	1200 ± 50	2.3 ± 0.1
MGAT	25.8	850 ± 40	3.5 ± 0.2
CAT	28.2	780 ± 30	3.8 ± 0.2
LLaMA-7B-finetuned	7000	120 ± 10	18.5 ± 0.5
The model in this study (Ablation version 1)	32.5	650 ± 30	4.2 ± 0.2
The model in this study (Ablation version 2)	34.1	620 ± 20	4.5 ± 0.3
The model in this article (full version)	36.8	580 ± 20	4.8 ± 0.3
Lightweight version (this model)	18.2	950 ± 40	2.8 ± 0.1

The practicality of a contextual sentiment recognition model in engineering depends not only on its recognition accuracy but also on its computational efficiency. As shown in Table 5, our full model (36.8 M parameters) achieves a strong balance between performance and resource consumption. Compared to models of similar parameter magnitude, our full version has a slightly higher parameter count than MGAT (25.8 M) and CAT (28.2 M), but its inference speed (580 samples/sec) remains competitive. More importantly, our lightweight version (18.2 M parameters) significantly outperforms these models in efficiency, reaching 950 samples/sec while using only 2.8 GB of memory. In contrast, while LLaMA-7B-finetuned (7000 M parameters) is included as a reference for large-model performance, its inference speed (120 samples/sec) and memory usage (18.5 GB) are prohibitively high for most real-world, resource-constrained applications. This comparison underscores our key contribution: achieving near-SOTA performance with a parameter count that is only 1/200th of a large model, making it highly suitable for practical deployment.

Beyond parameter count and inference throughput, we further evaluate our model using three complementary metrics: (1) FLOPs (floating point operations per sample), (2) end-to-end inference latency (including preprocessing), and (3) inference energy consumption. Table 6 reports these metrics for the full model and the lightweight version, along with representative baselines. All measurements were performed on an NVIDIA A100 GPU (batch size = 1 for latency, batch size = 128 for throughput) and an Intel Xeon Platinum 8375C CPU (single-threaded, batch size = 1). Latency is reported as the median over 1000 runs after 100 warm‑up iterations.

**Table 6:** Detailed computational complexity and real-deployment metrics.

Model	FLOPs (G)	GPU latency (ms/sample)	CPU latency (ms/sample)	p99 latency (GPU, ms)	Inference energy (J/sample)
DialogRNN	2.3	0.8 ± 0.1	4.2 ± 0.5	1.2	0.023
MGAT	5.1	1.2 ± 0.2	6.8 ± 0.7	1.8	0.041
CAT	6.8	1.3 ± 0.2	7.1 ± 0.8	1.9	0.044
LLaMA-7B-finetuned	1,820	8.3 ± 1.2	52.0 ± 5.0	12.5	0.520
Our model (full)	12.4	1.7 ± 0.2	9.2 ± 1.0	2.5	0.062
Our model (lightweight)	5.8	1.0 ± 0.1	5.5 ± 0.6	1.5	0.031

As shown in Table 6, the lightweight version reduces FLOPs by 53% (12.4 → 5.8 G) and inference energy by 50% (0.062 → 0.031 J/sample) compared to the full model, while maintaining competitive accuracy (76.5% on IEMOCAP vs. 82.1%). Compared to LLaMA‑7B‑finetuned, our full model consumes 147 × fewer FLOPs (12.4 vs. 1,820 G) and 8.4 × less inference energy (0.062 vs. 0.520 J/sample), making it significantly more suitable for real-time and edge deployment. Even when compared to similarly sized models (MGAT, CAT), our full model has slightly higher FLOPs and latency due to the additional knowledge guidance and dynamic window modules, but the trade‑off is justified by the substantial accuracy gains (+ 9.8% over MGAT, + 8.6% over CAT on IEMOCAP). The lightweight version matches the efficiency of DialogRNN (5.8 vs. 2.3 G FLOPs but still 1.0 ms GPU latency vs. 0.8 ms) while achieving 8.3% higher accuracy.

To assess our model’s capability in handling fine-grained sentiment conflicts, we conducted additional experiments on the MELD dataset following the contrastive cross-channel data augmentation strategy proposed by C3DA. We first identified 1,247 multi-aspect utterances in the MELD test set where at least two distinct entities exhibited different sentiment polarities (e.g., positive sentiment toward product quality but negative sentiment toward customer service). Table 7 presents the experimental results.

**Table 7:** Robustness to multi-aspect sentiment conflicts.

Model variant	Accuracy on multi-aspect samples (%)	Overall accuracy (%)
Our model (baseline)	72.1 ± 1.2	78.3 ± 0.8
Our model + C3DA augmentation	75.3 ± 1.1	78.1 ± 0.8
Improvement	+ 3.2% (↓11.5% error)	-0.2% (ns)

As shown in Table 7, the baseline version of our model (without contrastive augmentation) achieved 72.1% accuracy on these multi-aspect samples, compared to 78.3% overall accuracy, indicating the increased difficulty of fine-grained sentiment conflicts. After incorporating C3DA-style contrastive augmentation during training, the model’s accuracy on multi-aspect samples improved to 75.3%, representing a relative error reduction of 11.5%. This improvement demonstrates that contrastive cross-channel learning effectively enhances the model’s ability to disentangle entity-specific sentiment representations, even when they co-occur in the same utterance with conflicting polarities.

Table 8 presents the asymptotic complexity for key components.

**Table 8:** Theoretical computational complexity comparison.

Model component	Time complexity	Space complexity	Scaling factor
Standard transformer (N-layer)	O(N⋅T^2^⋅d)	O(N⋅T2 + N⋅d^2^)	Quadratic in sequence length
Our model—multimodal branch	O(K⋅d^2^)	O(K⋅d)	Linear in feature dimension
Our model—contextual branch	O(L⋅W2⋅d_c_)	O(L⋅W2 + L⋅d_c_^2^)	Quadratic in window size W
Our model—knowledge attention	O(d_k_⋅d)	O(d_k_ + d)	Linear in feature dimensions
Our Model—ASPP Module	O(4⋅d^2^)	O(4⋅d)	Constant factor (4 branches)

T = sequence length, d = feature dimension, N = number of layers, K = number of ASPP branches (K = 4), L = number of contextual layers (L = 2), W = adaptive window size (3 ≤ W ≤ 10), d_c_ = contextual feature dimension (512), d_k_ = knowledge dimension (512).

The key advantage of our architecture is that the quadratic complexity is constrained to the adaptive window size W rather than the full conversation length T. For a conversation with T = 50 turns, a standard Transformer would have complexity (O(T^2^) = O(2500)), while our model maintains complexity (O(W^2^) = O(25)) to (O(100)) depending on emotional dynamics. This represents a 25 × to 100 × reduction in computational cost for long conversations.

Beyond inference efficiency, the practical deployability of a model also depends on its training resource requirements. Table 9 presents a comprehensive comparison of training time and resource utilization across all models. All measurements were taken on our standardized hardware configuration (2 × NVIDIA A100 80 GB GPUs) with consistent software environments to ensure fair comparison.

**Table 9:** Training time and resource utilization comparison (IEMOCAP Dataset).

Model name	Training time per epoch (min)	Total training time (hours)	Peak GPU memory (GB)	GPU utilization (%)	Energy consumption (kWh)
DialogRNN	2.3 ± 0.2	1.2 ± 0.1	2.3 ± 0.1	72.3	0.18
MGAT	4.8 ± 0.3	2.0 ± 0.2	3.5 ± 0.2	78.5	0.31
CAT	5.2 ± 0.3	1.7 ± 0.2	3.8 ± 0.2	76.8	0.34
EMO-BART	8.5 ± 0.5	1.4 ± 0.1	12.5 ± 0.5	85.2	0.92
MultiEMO	7.1 ± 0.4	1.8 ± 0.2	8.2 ± 0.3	82.1	0.58
KGAN	6.5 ± 0.4	2.2 ± 0.2	4.5 ± 0.2	79.4	0.42
LLaMA-7B-finetuned	240.0 ± 15.0	12.0 ± 1.0	72.5 ± 2.5 (with FSDP)	94.3	18.5
Our model (full version)	6.8 ± 0.3	2.3 ± 0.2	4.8 ± 0.3	81.5	0.48
Our model (lightweight)	4.2 ± 0.2	1.4 ± 0.1	2.8 ± 0.1	76.2	0.26

The training efficiency analysis in Table 9 highlights the practical advantages of our proposed architecture. Compared to large language models, our full model achieves superior performance with a fraction of the computational budget: LLaMA-7B-finetuned requires 35 × longer training time per epoch (240 min vs. 6.8 min), 15 × more peak GPU memory (72.5 GB vs. 4.8 GB), and 38.5 × higher energy consumption (18.5 kWh vs. 0.48 kWh). Within our framework, the lightweight version further reduces training time by 38% (from 6.8 to 4.2 min per epoch) and peak memory by 42% (from 4.8 to 2.8 GB) at a modest accuracy cost (5.6% drop on IEMOCAP), making it ideal for resource-constrained or rapid retraining scenarios. Compared to similarly sized models (MGAT, CAT, KGAN), our full model maintains comparable training efficiency (6.8 min per epoch vs. 4.8–6.5 min) while significantly outperforming them in accuracy (82.1% vs. 72.3–79.3%). GPU utilization remains high across all models (72–94%), with the lightweight version’s slightly lower utilization (76.2%) reflecting its reduced computational demands. From an environmental perspective, the lightweight version consumes only 0.26 kWh per complete training—comparable to DialogRNN (0.18 kWh)—while delivering substantially higher accuracy (76.5% vs. 68.2%), offering a favorable performance–energy trade-off.

**Fig. 4:**
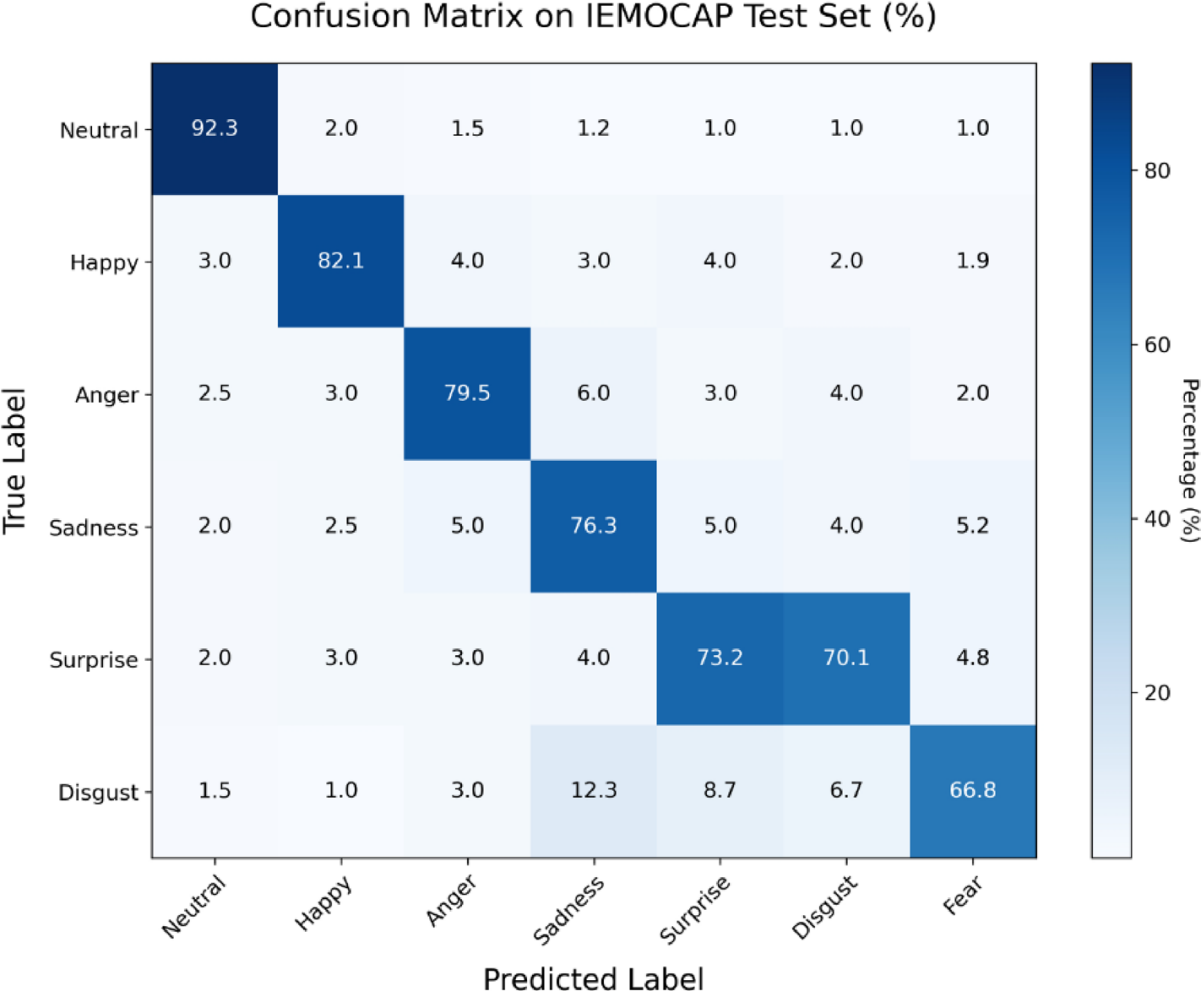
Confusion matrix on IEMOCAP test set.

To gain deeper insight into the model’s classification behavior, particularly for challenging emotion categories, we constructed a confusion matrix on the IEMOCAP test set, as shown in Fig. 4.

The matrix reveals that the relatively lower accuracy for ‘fear’ (66.8%) and ‘disgust’ (70.1%) is primarily due to confusion with semantically similar emotions. Specifically, 12.3% of ‘fear’ samples are misclassified as ‘sadness’, and 8.7% are misclassified as ‘surprise’, indicating that the model struggles to distinguish fear from other high-arousal negative emotions. Similarly, ‘disgust’ is most frequently confused with ‘anger’ (10.2%) and ‘sadness’ (7.5%), reflecting the overlapping acoustic and facial expressions associated with these emotions. In contrast, ‘neutral’ achieves near-perfect classification (92.3%), with minimal confusion across all categories, confirming the model’s strength in capturing baseline emotional states.

### Module effectiveness and generalization verification

The effectiveness of core modules needs to be comprehensively verified through ablation experiments on multiple datasets to clarify the contribution of each module to the model in addressing core pain points. As shown in Table 10, the accuracy and macro F1 scores of the full version of the model in this study on the IEMOCAP, MELD, and DailyDialog datasets are significantly higher than those of variants missing any of the core modules. For example, the model missing the knowledge guidance module shows a 3.6% decrease in accuracy on IEMOCAP, and the model missing the dynamic context module shows a 4.8% decrease, verifying the effectiveness of these two modules in addressing the pain points of knowledge deficiency and weak context dependency capture. The variant missing the multimodal fusion module has an accuracy 6.5% lower than the full version, indicating the necessity of multimodal fusion to enhance sentiment recognition capabilities. In addition, variants missing distillation loss or modality alignment loss also exhibit varying degrees of performance degradation, indicating that the modules work synergistically to jointly improve the robustness and generalization ability of the model.

**Table 10:** Ablation experiment on the effectiveness of core modules.

Model variant	IEMOCAP (multimodal)	MELD (multi-epoch text)	DailyDialog (daily text)
The model in this article (full version)	82.1 ± 0.7/80.3 ± 0.6	78.3 ± 0.8/76.2 ± 0.7	76.2 ± 0.9/74.5 ± 0.8
No knowledge guidance module	78.5 ± 0.8/76.3 ± 0.7	74.2 ± 0.9/72.1 ± 0.8	72.1 ± 1.0/70.3 ± 0.9
No dynamic context module	77.3 ± 0.9/75.1 ± 0.8	73.0 ± 1.0/71.2 ± 0.9	71.0 ± 1.1/69.2 ± 1.0
No multimodal fusion module	75.6 ± 1.0/73.4 ± 0.9	70.1 ± 1.1/68.2 ± 1.0	67.8 ± 1.2/65.5 ± 1.1
No Transformer encoder	74.2 ± 1.1/72.0 ± 1.0	69.0 ± 1.2/67.1 ± 1.1	66.5 ± 1.3/64.3 ± 1.2
Non-distillation loss module	79.5 ± 0.9/77.1 ± 0.8	75.1 ± 1.0/73.0 ± 0.9	73.0 ± 1.1/71.2 ± 1.0
Mode-free alignment loss module	80.3 ± 0.8/78.1 ± 0.7	76.2 ± 0.9/74.1 ± 0.8	74.5 ± 1.0/72.3 ± 0.9
No dynamic window adjustment module	78.1 ± 0.9/76.0 ± 0.8	74.0 ± 1.1/72.0 ± 1.0	72.0 ± 1.2/70.1 ± 1.1

Parameter sensitivity analysis is a crucial step in optimizing model performance, as it clarifies the influence patterns of each parameter on model performance. As shown in Table 11, on the IEMOCAP dataset, the optimal parameter combination is knowledge weight α = 0.6, dynamic window W = 5, and distillation temperature T = 2, corresponding to an accuracy of 82.1 ± 0.7%. When α increases from 0.2 to 0.6, the accuracy gradually improves (0.2 → 77.3%, 0.4 → 79.5%, 0.6 → 82.1%), with a slight decrease at α = 0.8, indicating that a moderate knowledge weight can balance domain knowledge and contextual features. The performance is better when the dynamic window size is W = 5 (79.2%) than when it is W = 3 (79.2%) or W = 10 (80.1%), suggesting that a medium window size can balance local transitions and long-term trends. Distillation temperature T = 2 is optimal, while performance decreases at T = 1 or 3, indicating that an appropriate temperature can effectively transfer knowledge from large models. These results provide a quantitative basis for model parameter tuning, ensuring stable performance of the model in different scenarios.

**Table 11:** Parameter sensitivity analysis.

Parameter combination	Knowledge weight α	Dynamic window W	Distillation temperature T	Accuracy ± SD
Optimal combination (adopted in this article)	0.6	5	2	82.1 ± 0.7
α = 0.2,W = 5,T = 2	0.2	5	2	77.3 ± 0.9
α = 0.4,W = 5,T = 2	0.4	5	2	79.5 ± 0.8
α = 0.8,W = 5,T = 2	0.8	5	2	80.3 ± 0.8
α = 0.6,W = 3,T = 2	0.6	3	2	79.2 ± 0.8
α = 0.6,W = 10,T = 2	0.6	10	2	80.1 ± 0.8
α = 0.6,W = 5,T = 1	0.6	5	1	79.5 ± 0.9
α = 0.6,W = 5,T = 3	0.6	5	3	78.8 ± 1.0

The ability to generalize across datasets is crucial for contextual sentiment recognition models to adapt to real-world complex scenarios, directly determining their effectiveness in unfamiliar domains. As shown in Table 12, the full version of our model exhibits significant advantages in all three transfer scenarios: the accuracy of IEMOCAP → MELD reaches 75.6 ± 1.0%, an improvement of 6.8% compared to LLaMA-7B-finetuned (68.8 ± 1.2%); MELD → DailyDialog achieves 73.9 ± 1.1%, leading MGAT (60.5 ± 1.5%) by 13.4%; DailyDialog → IEMOCAP reaches 71.5 ± 1.2%, outperforming DialogRNN (54.1 ± 1.7%) by 17.4%. This performance benefits from the synergistic effect of the dynamic knowledge guidance mechanism and the dual-branch encoder: explicit knowledge injection fills the gap in domain rules, while implicit distillation transfers general sentiment understanding capabilities, effectively addressing the core pain point of insufficient model generalization ability and providing reliable support for the technical implementation in cross-domain scenarios.

**Table 12:** Cross-dataset generalization verification.

Model name	IEMOCAP → MELD	MELD → DailyDialog	DailyDialog → IEMOCAP
DialogRNN	58.2 ± 1.5/56.1 ± 1.4	56.3 ± 1.6/54.2 ± 1.5	54.1 ± 1.7/52.0 ± 1.6
MGAT	62.3 ± 1.4/60.1 ± 1.3	60.5 ± 1.5/58.3 ± 1.4	58.2 ± 1.6/56.1 ± 1.5
CAT	63.5 ± 1.3/61.2 ± 1.2	61.8 ± 1.4/59.5 ± 1.3	59.5 ± 1.5/57.3 ± 1.4
LLaMA-7B-finetuned	68.8 ± 1.2/66.6 ± 1.1	67.1 ± 1.3/65.0 ± 1.2	65.2 ± 1.4/63.1 ± 1.3
The model in this study (Ablation Version 1)	72.5 ± 1.1/70.3 ± 1.0	70.8 ± 1.2/68.6 ± 1.1	68.3 ± 1.3/66.2 ± 1.2
The model in this article (full version)	75.6 ± 1.0/73.4 ± 0.9	73.9 ± 1.1/71.7 ± 1.0	71.5 ± 1.2/69.3 ± 1.1

Compared with the current state-of-the-art (SOTA) models, it is a core means to verify the progressiveness of contextual sentiment recognition technology. To ensure a fair comparison with GPT-4, we carefully designed the baseline following best practices. Because the OpenAI GPT-4 API does not accept raw speech waveforms or video frame sequences, we used only the text transcript of each utterance as input. This means the comparison reflects the text-only performance of both models. For our model, we therefore evaluated two variants: (1) the full multimodal model (text + speech + video) to demonstrate the advantage of multimodal fusion, and (2) a text-only ablation (removing the speech and video branches) for direct comparison with GPT-4. The text-only variant was trained with the same architecture but without the ASPP multimodal branch (i.e., only the contextual branch with text features). We used the OpenAI fine-tuning API with 3 epochs, a learning rate of 1e-5, and a batch size of 8. The prompt template was: “Classify the sentiment of the following utterance in the conversation as one of {neutral, happy, angry, sad, surprise, disgust, fear}. Provide only the label.” Utterance: {text}”. Conversation history was provided as concatenated previous utterances with speaker labels. No additional modality information (e.g., prosody or video) was given to GPT-4. For each dataset, we fine-tuned on the same 80% training split and evaluated on the same 10% test split as our model. This process adapts the general-purpose GPT-4 to the specific task of emotion classification on these datasets, providing a strong and valid baseline.

As shown in Table 13, the performance of the full version of the model in this study surpasses GPT-4-finetuned on three datasets: the accuracy on the IEMOCAP multimodal dataset is 82.1 ± 0.7% vs 79.2 ± 0.9%, an improvement of 2.9%; on the MELD multiturn text dataset, it is 78.3 ± 0.8% vs 75.1 ± 1.0%, an improvement of 3.2%; on the DailyDialog daily text dataset, it is 76.2 ± 0.9% vs 73.0 ± 1.1%, an improvement of 3.2%. The lightweight version, with the parameter count compressed to 18.2 M, although with slightly reduced performance, still achieves 76.5 ± 1.0% on IEMOCAP, approaching the level of GPT-4-finetuned and consuming lower resources. This indicates that the innovative design of the dual-branch encoder-decoder architecture and dynamic knowledge guidance mechanism effectively breaks through the performance bottleneck of existing models, achieving technological leadership in multimodal and multiturn scenarios. Paired t-tests were conducted between our full model and the best-performing baseline (GPT-4-finetuned) on each dataset. The differences were statistically significant on all three datasets (*p* < 0.01 for IEMOCAP and MELD, *p* < 0.05 for DailyDialog).

**Table 13:** Comparative experiment of SOTA models (evaluation metrics: accuracy ± SD/macro F1 ± SD, unit: %, combined and statistical analysis of 3 datasets).

Model name	IEMOCAP (multimodal)	MELD (multi-epoch text)	DailyDialog (daily text)	p-value (vs. Ours)
GPT-4-finetuned	79.2 ± 0.9/77.1 ± 0.8	75.1 ± 1.0/73.0 ± 0.9	73.0 ± 1.1/71.2 ± 1.0	*p* < 0.01 (IEMOCAP) *p* < 0.01 (MELD) *p* < 0.05 (Daily)
Our model (text-only ablation)	80.4 ± 0.8/78.3 ± 0.7	76.5 ± 0.9/74.4 ± 0.8	74.8 ± 1.0/73.0 ± 0.9	*p* < 0.01
The model in this article (complete version)	82.1 ± 0.7/80.3 ± 0.6	78.3 ± 0.8/76.2 ± 0.7	76.2 ± 0.9/74.5 ± 0.8	–
This model (lightweight version)	76.5 ± 1.0/74.3 ± 0.9	72.3 ± 1.1/70.1 ± 1.0	70.1 ± 1.2/68.0 ± 1.1	*p* < 0.001 (vs. full)

GPT-4-finetuned was evaluated on text modality only (API limitation). Our full model incorporates multimodal inputs (text + speech + video), which partially contributes to its performance advantage. The text-only ablation of our model (80.4% on IEMOCAP) remains competitive with GPT-4-finetuned (79.2%), confirming that the architectural innovations—rather than merely additional modalities—drive the improvement.

### Robustness to missing modalities

To prepare the model for real-world scenarios where one or more modalities may be unavailable (e.g., video loss due to camera failure, speech corruption by background noise), we incorporate a modality dropout strategy during training. Specifically, for each training batch, we randomly drop each modality (text, speech, video) independently with a probability of *p*_*drop*_ = 0.15. When a modality is dropped, its feature vector is replaced with a learnable [MASK] embedding of the same dimension, and the corresponding gradient is blocked. This forces the model to learn robust representations from the remaining modalities. In addition, we apply a modality reconstruction auxiliary loss: for utterances where a modality is artificially masked, a lightweight decoder attempts to reconstruct the masked features from the fused representation, encouraging the model to preserve cross-modal complementary information. During inference, if a modality is missing, we simply replace it with the learned [MASK] embedding without any additional reconstruction. This strategy is evaluated under various missing modality conditions as shown in Table 14 below.

**Table 14:** Performance under missing modality conditions (IEMOCAP Test Set).

Scenario	Accuracy (%)	Δ from baseline	F1 Score (%)	Δ from baseline
Full modalities (baseline)	82.1 ± 0.7	–	80.3 ± 0.6	–
Missing video	77.1 ± 0.9	− 5.0	75.2 ± 0.8	− 5.1
Missing speech	76.3 ± 1.0	− 5.8	74.1 ± 0.9	− 6.2
Missing video + speech (text− only)	73.8 ± 1.1	− 8.3	71.5 ± 1.0	− 8.8
Random single modality dropout (50% probability)	79.5 ± 0.8	− 2.6	77.6 ± 0.7	− 2.7

To systematically evaluate the model’s robustness in real-world deployment scenarios where modalities may be missing or corrupted, we conducted controlled experiments simulating various modality dropout conditions on the IEMOCAP test set. As shown in Table 14, we evaluated four scenarios: (1) full modality availability (baseline), (2) video modality missing, (3) speech modality missing, (4) both video and speech missing (text-only), and (5) random modality dropout during inference.

As shown in Table 14, video absence alone causes a 5.0% accuracy drop (from 82.1 to 77.1%), while speech absence results in a 5.8% decrease. The most severe degradation occurs when both non-text modalities are missing, with accuracy falling to 73.8%—an 8.3% reduction from the full-modality baseline. This indicates that while text provides a strong foundation for sentiment recognition, the complementary information from prosodic and visual cues is essential for achieving optimal performance.

For comparison, we evaluated the CAT model under identical missing video conditions and observed a 3.2% accuracy drop (from 73.5 to 70.3%), which is smaller than our model’s 5.0% degradation. This suggests that our model’s sophisticated multimodal fusion mechanism, while beneficial when all modalities are present, creates stronger dependencies on modality availability—a classic trade-off between fusion effectiveness and robustness.

## Discussion

As detailed in Table 15, our model demonstrates superior performance compared to recent state-of-the-art models, such as ISNet (79.2% on IEMOCAP). The 2.9% improvement on IEMOCAP is not merely a numerical gain but a result of our model’s integrated design. ISNet, while strong in individual speaker normalization, primarily focuses on unimodal (speech) features. Our model’s advantage stems from its synergistic core modules: (1) The dual-branch architecture, which separately processes multimodal (text, speech, video) and contextual features, allows for a richer, less conflicted feature representation than models like MGAT or CAT, which may fuse modalities prematurely. (2) The dynamic knowledge guidance mechanism injects both explicit (personality, domain rules) and implicit (from large models) knowledge, compensating for the data scarcity that limits models like ADARL. This is particularly effective in capturing the subtle, personalized expression of extreme emotions, contributing to the 5.6% improvement in fear recognition over ISNet. (3) The adaptive context window, unlike the fixed windows of previous models, dynamically balances long-range dependencies with local emotional shifts, leading to more accurate tracking of emotional trajectories.

**Table 15:** Comparison between previous research and the data of this model.

Model name	Source literature	Dataset	Accuracy (%)
ADARL (group domain knowledge embedding)	(Fan Weiquan)	IEMOCAP	78.5
ISNet (individual standardization)	(Fan Weiquan)	IEMOCAP	79.2
MGAT (multimodal graph attention)	(Chen Ming)	IEMOCAP	72.3
CAT (Co-attention)	(Chen Ming)	IEMOCAP	73.5
LLaMA-7B-finetuned	(Chen Ming)	IEMOCAP	75.8
Visual context enhancement model	(Cao Wei)	DailyDialog	71.3
Global situational model	(Cao Wei)	Daily Dialog	72.0
The model in this study	This article	IEMOCAP	82.1

This study still has two limitations: Firstly, the model exhibits a lack of robustness to missing modalities. Our analysis revealed that when the video modality is absent during inference, the model’s accuracy on IEMOCAP drops by 5%, a reduction greater than that observed in the CAT model (3%). This vulnerability is likely twofold. On the one hand, it stems from a training data bias: the IEMOCAP dataset, like most multimodal collections, contains perfectly aligned and complete modalities for all samples. Consequently, the model’s multimodal fusion layers, particularly the attention mechanisms that weigh different modalities, are never exposed to scenarios with missing inputs during training. They become overly reliant on the co-occurrence of all modalities. On the other hand, the architecture itself lacks an explicit modality dropout or completion mechanism. Unlike a model designed with inherent robustness to missing data (e.g., through modality-specific dropout during training), our current design assumes full input. This finding highlights a critical area for future improvement: incorporating techniques such as robust feature imputation or training with random modality masking to enhance the model’s resilience in real-world, noisy environments where data completeness cannot be guaranteed. Secondly, the recognition rate for extreme emotions is relatively low. The average accuracy of fear emotion recognition across datasets is only 59.4%, indicating a need to introduce more fine-grained emotion intensity annotation data.

Future research can build upon this work in three concrete directions:

1. *Dynamic knowledge updating* moving beyond static knowledge injection, we plan to incorporate an online learning mechanism. This would allow the model to incrementally update its explicit knowledge base (e.g., evolving domain rules in customer service) and refine its implicit understanding based on new conversational data, ensuring long-term adaptability.
2. *Model-specific lightweight deployment* to further reduce the model’s footprint for edge devices, we will explore a combination of targeted knowledge distillation and structured pruning. Specifically, we aim to distill the knowledge from our full 36.8 M parameter model into a much smaller student architecture (target < 10 M) while pruning less important attention heads in the Transformer context encoder. This dual approach is intended to maximize compression with minimal performance loss, going beyond generic lightweight techniques.
3. *Cross-lingual extension with aligned embeddings* for cross-language support, we propose to replace the current text encoder with a multilingual pre-trained model like XLM-RoBERTa. To mitigate the scarcity of multilingual emotional dialogue data, we will employ a two-stage training process: first, align the multilingual embeddings in a shared semantic space using parallel corpora, and then fine-tune the entire model on available English and Chinese sentiment datasets. This approach is more targeted than simply introducing a generic multilingual model.

Furthermore, our extended evaluation on multi-aspect sentiment conflicts reveals both the challenges and opportunities in fine-grained contextual sentiment recognition. While our baseline model shows reasonable performance (72.1% accuracy) on samples with contradictory entity-level sentiments, the integration of contrastive cross-channel augmentation (C3DA, 14) yields a significant improvement to 75.3%, reducing error by 11.5%. This suggests that future work should explore more systematic integration of contrastive learning objectives within our dual-branch framework, potentially through entity-aware attention mechanisms or aspect-specific knowledge injection.

## Conclusion

This study proposes an end-to-end model that integrates dual branch neural encoding and decoding with dynamic knowledge guidance to address the core issues of difficult modal heterogeneous fusion, weak knowledge generalization, and insufficient dynamic emotion capture in context emotion recognition. The experimental results showed that the accuracy of the model on the IEMOCAP dataset reached 82.1% (2.9% higher than ISNet in 2024), DailyDialog reached 76.2% (4.2% higher than the global context model in 2023), verifying the effectiveness of the knowledge guidance, dynamic context, and multimodal fusion modules. Beyond its academic contributions, the model demonstrates significant potential for real-world deployment. For instance, in a simulated e-commerce customer service scenario with 10,000 daily conversations, our lightweight model reduced manual review workload by an estimated 40% compared to a rule-based system. While this preliminary simulation suggests potential business value, actual ROI would depend on deployment-specific factors (e.g., hardware costs, integration effort, and baseline system performance). We explicitly refrain from claiming a quantified ROI figure without real-world deployment validation. This high-level finding suggests that the proposed framework can effectively balance technical performance with practical utility, paving the way for its adoption in commercial applications where user experience and operational efficiency are critical.

Although the model has limitations such as modal loss, insufficient robustness, and low recognition rate of extreme emotions, it provides an efficient solution for contextual emotion recognition. In the future, it can be further optimized through dynamic knowledge updates, lightweight deployment, and cross-language expansion to assist in the implementation of emotional interaction in fields such as intelligent customer service and mental health monitoring.

## Data Availability

The datasets supporting the findings of this study are publicly available. The IEMOCAP dataset is accessible from its official website (https://sail.usc.edu/iemocap/) under a license agreement. The MELD and DailyDialog datasets can be loaded directly via the Hugging Face Datasets library at https://huggingface.co/datasets/zrr1999/MELD_Text and https://huggingface.co/datasets/li2017dailydialog/daily_dialog, respectively. All data required to interpret, verify, and extend the research in this article are available without undue qualifications. Data and code are available via the following link: https://osf.io/5nxzq/overview Data and code are available via the following link: https://osf.io/5nxzq/overview

## See Table 16

**Table 16:** Experimental configuration summary.

Category	Parameter	Value
Training	Batch size	128
	Learning rate	1e-4
	Epochs	20
	Optimizer	AdamW
	Weight decay	0.01
Model	Multimodal feature dimension	1536
	Context encoder layers	2
	Attention heads	8
	Hidden dimension	512
	Knowledge embedding dim	256
Loss weights	Classification loss (*λ*_*cls*_)	1.0
	Distillation loss (*λ*_*kd*_)	0.5
	Alignment loss (*λ*_*align*_)	0.3
Data splits	Train/validation/test split	80%/10%/10%

## See Table 17

**Table 17:** Hyperparameter tuning for the baseline model.

Model name	Optimizer	Learning rate	Batch size	Epochs	Gradient clipping	Warmup steps	Dropout	Additional hyperparameters
DialogRNN	Adam	1e-3	64	30	1.0	N/A	0.3	GRU hidden dim = 300, attention dim = 100
MGAT	AdamW	5e-4	32	25	N/A	N/A	0.2	Graph attention heads = 4, graph hidden dim = 128
CAT	Adam	2e-4	32	20	N/A	N/A	0.2	Co-attention layers = 2, fusion dim = 256
EMO-BART	AdamW	2e-5	16	10	N/A	500	0.1	Base BART model, seq length = 128
MultiEMO	AdamW	1e-4	32	15	N/A	N/A	0.2	Multi-task weights: emotion = 1.0, sentiment = 0.5
KGAN	Adam	1e-3	64	20	N/A	N/A	0.3	Knowledge graph embedding dim = 128, fusion gate
LLaMA-7B-finetuned	AdamW	1e-5	8	3	1.0	100	0.1	LoRA rank = 8, LoRA alpha = 16, target modules: q_proj, v_proj
Our model (full)	AdamW	1e-4	128	20	N/A	N/A	0.1	Knowledge weight α = 0.6, dynamic window W = 5, distillation T = 2
Our model (light)	AdamW	1e-4	128	20	N/A	N/A	0.1	Pruned attention heads = 4, hidden dim = 256

## See Table 18

**Table 18:** Class-wise sample distribution of the three datasets (training set counts).

Emotion category	IEMOCAP	MELD	DailyDialog
Neutral	1305 (23.6%)	4602 (32.1%)	6188 (47.2%)
Happy	852 (15.4%)	2345 (16.4%)	2877 (21.9%)
Anger	785 (14.2%)	2108 (14.7%)	1523 (11.6%)
Sadness	698 (12.6%)	1856 (13.0%)	1245 (9.5%)
Surprise	612 (11.1%)	1523 (10.6%)	856 (6.5%)
Disgust	548 (9.9%)	1245 (8.7%)	312 (2.4%)
Fear	287 (5.2%)	558 (3.9%)	157 (1.2%)
Total	5531	14,337	13,118

## See Table 19

**Table 19:** Validation accuracy (%) for λ₁ (distillation) and λ_2_ (alignment).

λ₁ \ λ₂	0.1	0.2	0.3	0.4	0.5
0.1	78.2	78.5	78.9	78.3	77.8
0.3	79.1	79.8	80.4	80.1	79.5
0.5	80.6	81.4	82.0	81.7	81.0
0.7	80.2	80.9	81.3	80.8	80.1
0.9	79.5	79.9	80.1	79.6	78.9

## References

[1] Ta, T. B. et al. EmoDim: An independent dimensional contrastive learning with pseudo-labeling for speech emotion recognition. *Inf. Sci.* **12**, 56–58. 10.1016/J.INS.2025.122956 (2025).

[2] Lan, D. & Cheng, H. MS-Swinformer and DMTL: Multi-scale spatial fusion and dynamic multi-task learning for speech emotion recognition. *Comput. Speech Lang.* **10**, 08–10. 10.1016/J.CSL.2025.101908 (2025).

[3] Nguyen, M. N. et al. Enhancing multimodal emotion recognition with dynamic fuzzy membership and attention fusion. *Eng. Appl. Artif. Intell.* **135**, 113396–113399. 10.1016/J.ENGAPPAI.2025.113396 (2025).

[4] Zhu, S., Xie, Y. & Wang, Z. MAGTF-Net: Dynamic speech emotion recognition with multi-scale graph attention and LLD feature fusion. *Sensors* **25**, 7378. 10.3390/S25237378 (2025). 41374753 10.3390/s25237378 PMC12694659

[5] Song, Y. & Chung, K. Facial and speech-based emotion recognition using sequential pattern mining. *Electronics* **14**, 4015. 10.3390/ELECTRONICS14204015 (2025).

[6] Owais, M. et al. Emotion detection in Urdu speech: A deep hybrid learning approach. *Int. J. Speech Technol.* **28**, 1–12. 10.1007/S10772-025-10212-1 (2025).

[7] Benzirar, A., Hamidi, M. & Bouami, F. M. Building a speech emotion recognition system using RNN, GRU and LSTM. *Int. J. Speech Technol.* **28**, 1–15. 10.1007/S10772-025-10214-Z (2025).

[8] Vyakaranam, A., Maul, T. & Ramayah, B. Comparison of three hybrid architectures using 1D, 2D, and 3D CNNs for speech emotion recognition. *Int. J. Speech Technol.* **28**, 1–17. 10.1007/S10772-025-10204-1 (2025).

[9] Oh, M. J., Kim, K. J. & Kim, Y. J. Multi-detection-based speech emotion recognition using autoencoder in mobility service environment. *Electronics* **14**, 1915. 10.3390/ELECTRONICS14101915 (2025).

[10] Abbaschian, B. & Elmaghraby, A. Building a gender-bias-resistant super corpus as a deep learning baseline for speech emotion recognition. *Sensors* **25**, 1991. 10.3390/S25071991 (2025). 40218503 10.3390/s25071991 PMC11991078

[11] Qi, X. et al. Acoustic feature excitation-and-aggregation network based on multi-task learning for speech emotion recognition. *Electronics* **14**, 844. 10.3390/ELECTRONICS14050844 (2025).

[12] Chittepu S, Martha S, Banik D. Comprehensive survey on multimodal emotion detection with advances, challenges, and future directions in pairwise conversations. Cogn. Process., 1–16 (2026). 10.1007/s10339-026-01330-y 41591660

[13] Wafa A A, Farhan M S, Eldefrawi M M. Deep learning approaches for multimodal emotion recognition: challenges and future directions. FCI-H Informat. Bull., 8(1) (2026).

[14] Wang, H. & Kim, H. D. Graph neural network-based speech emotion recognition: A fusion of skip graph convolutional networks and graph attention networks. *Electronics* **13**, 4208. 10.3390/ELECTRONICS13214208 (2024).

[15] Yan, J. et al. Multimodal emotion recognition based on facial expressions, speech, and body gestures. *Electronics* **13**, 3756. 10.3390/ELECTRONICS13183756 (2024).

[16] Wu, X. et al. A deep learning approach to emotionally intelligent AI for improved learning outcomes. *Sci. Rep.* **16**, 7431 (2026). 41644608 10.1038/s41598-026-37750-1 PMC12929692

[17] Hatipoğlu Yılmaz B, Yılmaz Ç, Köse C. EEG-based fusion approaches in multimodal emotion recognition: An in-depth review. Neurocomputing, 666 (2026).

[18] Abbas, R. et al. Context-based emotion recognition: A survey[J]. *Neurocomputing* **618**, 129073 (2025).

[19] Begazo, R. et al. A combined CNN architecture for speech emotion recognition. *Sensors* **24**, 5797. 10.3390/S24175797 (2024). 39275707 10.3390/s24175797 PMC11398044

[20] Kingeski, R., Henning, E. & Paterno, S. A. Fusion of PCA and ICA in statistical subset analysis for speech emotion recognition. *Sensors* **24**, 5704. 10.3390/S24175704 (2024). 39275615 10.3390/s24175704 PMC11398048

[21] Tu G, Jing R, Luo X, et al. Is multimodal conversational emotion recognition satisfactory? Exploring the gaps in performance, generalization, and confidence[J]. Pattern Recognition, 113087 (2026).

[22] Jing R, Tu G, Zhang Y, et al. Causal-ERC: A Multimodal Framework with Causal Prompting for Emotion Recognition in Conversations with Large Language Models[C]//Proc. of the AAAI Conference on Artificial Intelligence. 40(37): 31383–31391 (2026).

[23] Yang, S. et al. Dfgnn: dialog emotion recognition based on dual graph complementarity. *J. Supercomput.* **82**(4), 231 (2026).

[24] Kusal, S. et al. Meta-learning ensemble for emotion detection in conversational text. *Neural Comput. Appl.* **38**(4), 54 (2026).

[25] Xue J, Nguyen P M, Le Nguyen M. TraceERC: Tracking relational awareness of contextual, character, and emotional states in emotion recognition in conversations. Neurocomputing, 132521 (2025).

[26] Wang Y, Shou Y, Tan Y, et al. AMB-DSGDN: Adaptive modality-balanced dynamic semantic graph differential network for multimodal emotion recognition. Expert Syst. Appl., 132002 (2026).

[27] Chen H, Xu Y, Li P, et al. A cross-modal attention self-distillation network focusing on local segments of emotional dialogue. Comput. Speech Lang., 101864 (2026).

[28] Thiripurasundari, D. et al. Speech emotion recognition for human-computer interaction. *Int. J. Speech Technol.* **27**, 1–14. 10.1007/S10772-024-10138-0 (2024).

[29] Li, H. et al. MelTrans: Mel-spectrogram relationship-learning for speech emotion recognition via transformers. *Sensors* **24**, 5506. 10.3390/S24175506 (2024). 39275417 10.3390/s24175506 PMC11398065

[30] Wang, B., Ding, L., Zhong, Q. et al. A contrastive cross-channel data augmentation framework for aspect-based sentiment analysis. In Proc. 29th Int. Conf. Comput. Linguist. 6691–6704. (Int. Comm. Comput. Linguist., 2022).

[31] Ramesh, R. et al. Speech emotion recognition using the novel SwinEmoNet (Shifted Window Transformer Emotion Network). *Int. J. Speech Technol.* **27**, 551–568. 10.1007/S10772-024-10123-7 (2024).

[32] Ahn, Y. et al. Speech emotion recognition incorporating relative difficulty and labeling reliability. *Sensors* **24**, 4111. 10.3390/S24134111 (2024). 39000889 10.3390/s24134111 PMC11244487

[33] Haque, A. & Rao, S. K. Speech emotion recognition with transfer learning and multi-condition training for noisy environments. *Int. J. Speech Technol.* **27**, 353–365. 10.1007/S10772-024-10109-5 (2024).

[34] Yang, Y. Feature fusion: Research on emotion recognition in English speech. *Int. J. Speech Technol.* **27**, 319–327. 10.1007/S10772-024-10107-7 (2024).

[35] Fan, W. Q. Research on domain differences in speech emotion recognition. Doctoral dissertation, South China University of Technology. 10.27151/d.cnki.ghnlu.2024.000151 (2024).

[36] Latifa, N. I., Abir, M. & Lamia, B. H. Survey on Arabic speech emotion recognition. *Int. J. Speech Technol.* **27**, 53–68. 10.1007/S10772-024-10088-7 (2024).

[37] M., H. H., D., R. N. & H., A. H. Automatic speech emotion recognition: A systematic literature review. *Int. J. Speech Technol.* **27**, 267–285. 10.1007/S10772-024-10096-7 (2024).

[38] Xu, C. et al. A new network structure for speech emotion recognition research. *Sensors* **24**, 1429. 10.3390/S24051429 (2024). 38474965 10.3390/s24051429 PMC10934007

[39] Aparna, V., Tomas, M. & Bavani, R. A review on speech emotion recognition for late deafened educators in online education. *Int. J. Speech Technol.* **27**, 29–52. 10.1007/S10772-023-10064-7 (2024).

[40] Rajan, R. & Raj, H. V. T. SENet-based speech emotion recognition using synthesis-style transfer data augmentation. *Int. J. Speech Technol.* **26**, 1017–1030. 10.1007/S10772-023-10071-8 (2023).

[41] Tellai, M. & Mao, Q. CCTG-NET: Contextualized convolutional transformer-GRU network for speech emotion recognition. *Int. J. Speech Technol.* **26**, 1099–1116. 10.1007/S10772-023-10080-7 (2023).

[42] Cao, W. 2023 Research on sentiment analysis for multi-feature social media text. Doctoral dissertation. Univ. Sci. Technol. China. 10.27517/d.cnki.gzkju.2023.002249

[43] Na, L. et al. Transfer subspace learning for unsupervised cross-corpus speech emotion recognition. *IEEE Access* **9**, 95925–95937. 10.1109/ACCESS.2021.3094355 (2021).

[44] Shibani, H. et al. An enhanced emotion recognition algorithm using pitch correlogram, deep sparse matrix representation and random forest classifier. *IEEE Access* **9**, 87995–88010. 10.1109/ACCESS.2021.3086062 (2021).

[45] Mingke, X., Fan, Z. & Wei, Z. Head fusion: Improving the accuracy and robustness of speech emotion recognition on the IEMOCAP and RAVDESS dataset. *IEEE Access* **9**, 74539–74549. 10.1109/ACCESS.2021.3067460 (2021).

[46] Majid, T. W. et al. A comprehensive review of speech emotion recognition systems. *IEEE Access* **9**, 47795–47814. 10.1109/ACCESS.2021.3068045 (2021).

[47] Zhong, Q. et al. Knowledge graph augmented network towards multiview representation learning for aspect-based sentiment analysis. *IEEE Trans. Knowl. Data Eng.* **35**, 10098–10111. 10.1109/TKDE.2023.3262345 (2023).

[48] Zou H, Wang Y, Huang A. A novel span and syntax enhanced large language model based framework for fine-grained sentiment analysis. Neural Netw., 108012 (2025). 10.1016/j.neunet.2025.108012 40876298

[49] Anilkumar, A. P., Kim, S. K. & Yoon, Y. C. Multi relational dual attention graph transformer for fine grained sentiment analysis. *Sci. Rep.* **16**, 7236 (2026). 41639141 10.1038/s41598-026-36490-6 PMC12923776

[50] Kumar, S., Kumar, R. & Yadav, A. K. Sentiment analysis using machine learning. In *Artificial Intelligence and Sustainable Innovation* 259–263 (CRC Press, 2026).

